# Supplementary material for: Hormetic heat stress and HSF-1 induce autophagy to improve survival and proteostasis in C. elegans
Source: Nat Commun. 2017 Feb 15;8:14337. doi: 10.1038/ncomms14337 (PMC5316864; doi:10.1038/ncomms14337)
Supplement: Supplementary Information — Supplementary Figures, Supplementary Tables and Supplementary Reference. [file ncomms14337-s1.pdf]

1. Supplementary Figures and Legends

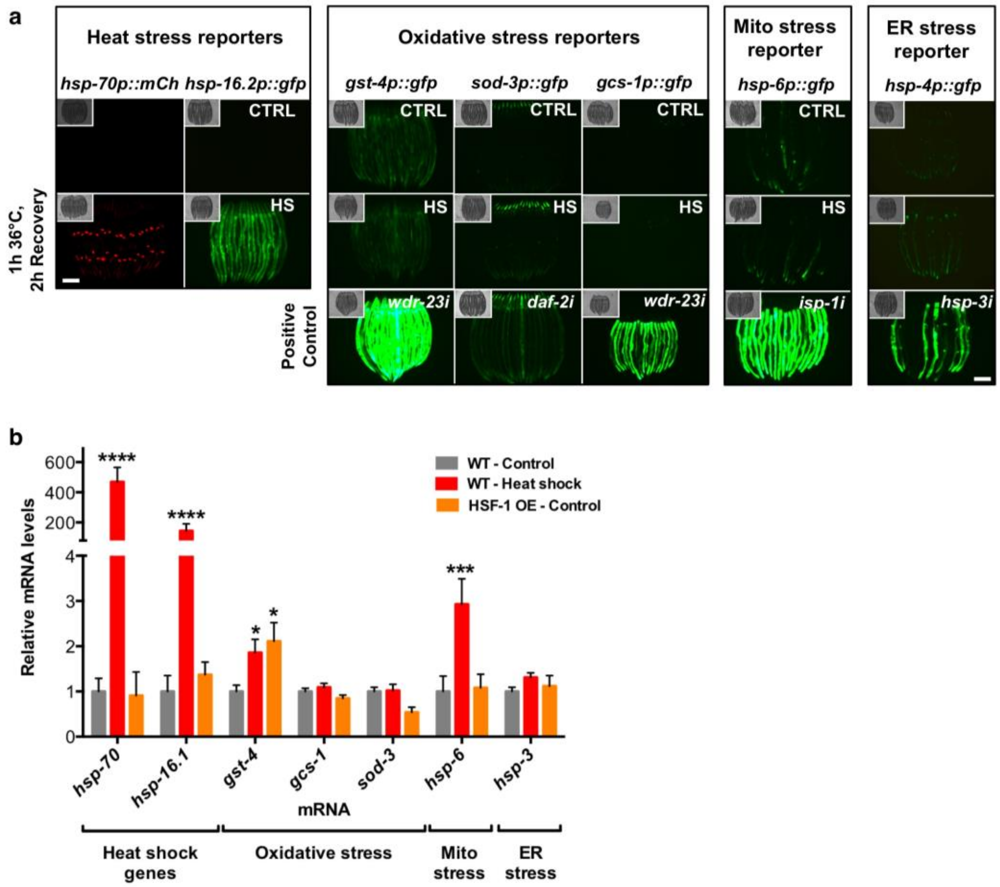

Supplementary Figure 1. Hormetic heat shock specifically induces the HSR. (**a**) mCherry and GFP expression was assessed in the indicated stress reporter strains, which were maintained under control conditions (CTRL, upper row), subjected to 1 h of heat shock at 36°C (HS) followed by 2 h recovery (HS, middle row), or raised on bacteria expressing dsRNA targeting *wdr-23*, *daf-2*, *isp-1,* or *hsp-3* to induce the indicated stress response (lower row). Exposure time: 1000 ms. Scale bar: 200 µm. The experiment was performed twice with similar results. Inserts show bright-field images of the same animals. (**b**) Transcript levels of genes involved in various stress responses in wild-type (WT, N2) and HSF-1–overexpressing (HSF-1 OE) animals maintained under control conditions (CTRL) or in WT animals subjected to heat shock for 1 h at 36°C. Data are the mean ± s.e.m. of four biological replicates, each with three technical replicates, and are normalized to the mean expression levels of four housekeeping genes. *$P < 0.05$, ***$P < 0.001$, ****$P < 0.0001$, no symbol: $P > 0.05$ by one-way ANOVA per transcript.

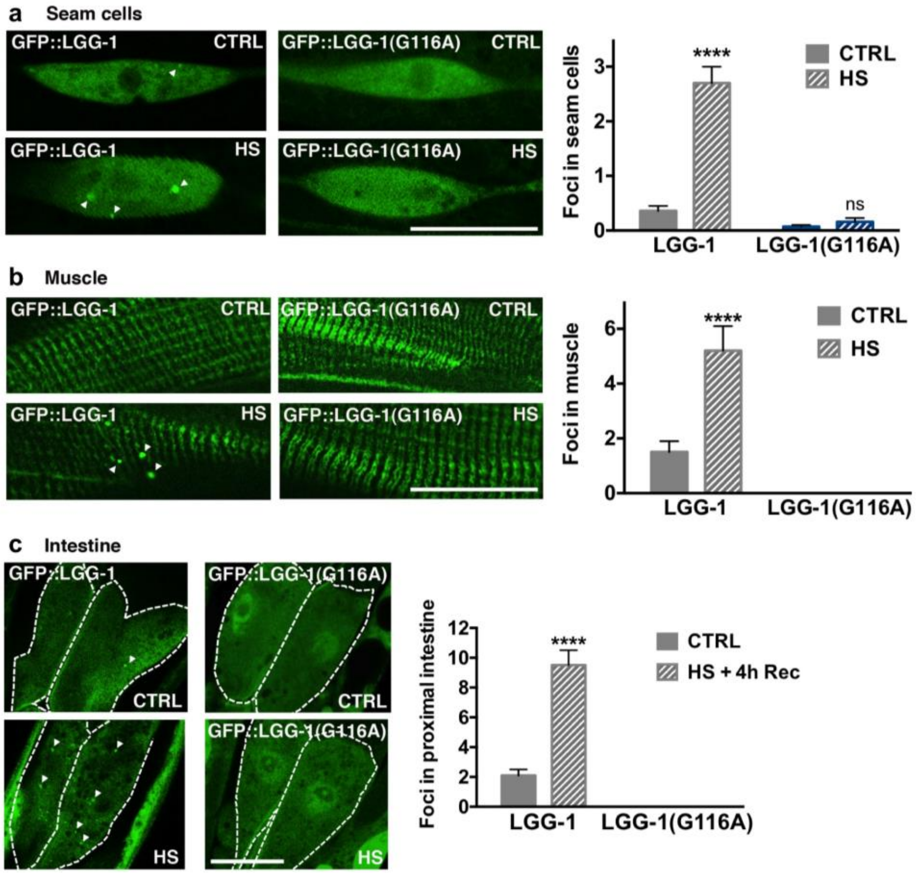

Supplementary Figure 2. The heat shock-mediated increase in GFP::LGG-1-positive punctae requires LGG-1 lipidation. GFP::LGG-1/Atg8 punctae were counted on day 1 of adulthood in *C. elegans* expressing *lgg-1p::gfp::lgg-1* (LGG-1) or *lgg-1p::gfp::lgg-1(G116A)* (LGG-1(G116A)) and either maintained under control conditions (CTRL) or subjected to heat shock for 1 h at 36°C (HS) followed by the indicated recovery period (Rec). Punctae were examined in (**a**) hypodermal seam cells (N = 45-60 cells), (**b**) body-wall muscle (N=10-11 animals), and (**c**) proximal intestinal cells (N = 13-14 animals). See also Supplementary Table 4 for a summary of repeat experiments. Error bars indicate s.e.m. Scale bar: 20 μm. ns: $P > 0.05$, ****$P < 0.0001$ by multiple *t*-tests.

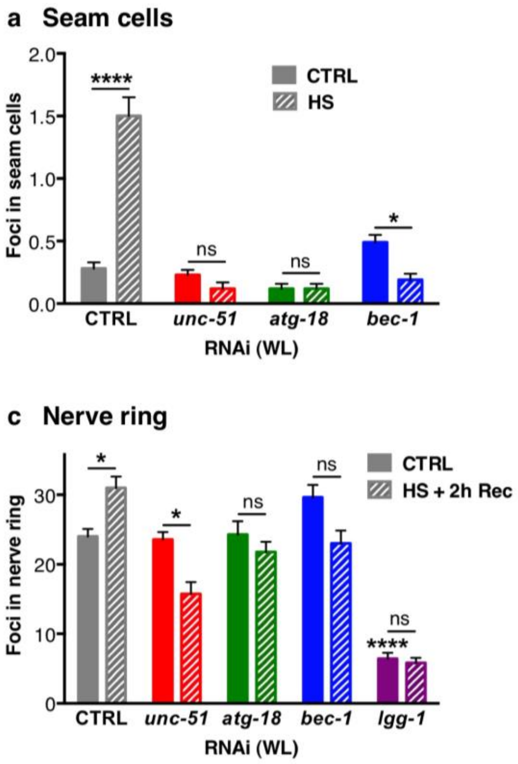

Supplementary Figure 3. Induction of autophagy by hormetic heat shock requires autophagy genes. GFP::LGG-1/Atg8 punctae were counted on day 1 of adulthood in *C. elegans* expressing (**a,b,d**) *lgg-1p::gfp::lgg-1* or (**c**) *rgef-1p::gfp::lgg-1* maintained under control conditions (CTRL, solid bars) or subjected to heat shock for 1 h at 36°C (HS, striped bars) followed by the indicated recovery period (Rec). Animals were raised from hatching (WL: whole-life RNAi) on control bacteria (empty vector, CTRL) or bacteria expressing dsRNA targeting the autophagy genes *unc-51/ATG1*, *atg-18, bec-1/ATG6,* or *lgg-1/ATG8.* Punctae were examined in (**a**) hypodermal seam cells (N = 64-100 cells), (**b**) body-wall muscle (N = 11-13 animals), (**c**) nerve ring neurons (N = 11-13 animals), and (**d**) intestinal cells (N = 12-13 animals). Although neurons are partially refractory to RNAi, GFP::LGG-1/Atg8 punctae in neurons were reduced after *lgg-1* RNAi, indicating that neurons are at least partially able to process RNAi. Error bars indicate s.e.m. ns: $P > 0.05$, $*P < 0.05$, $****P < 0.0001$ by two-way ANOVA. See also Supplementary Table 5 for a summary of additional experiments.

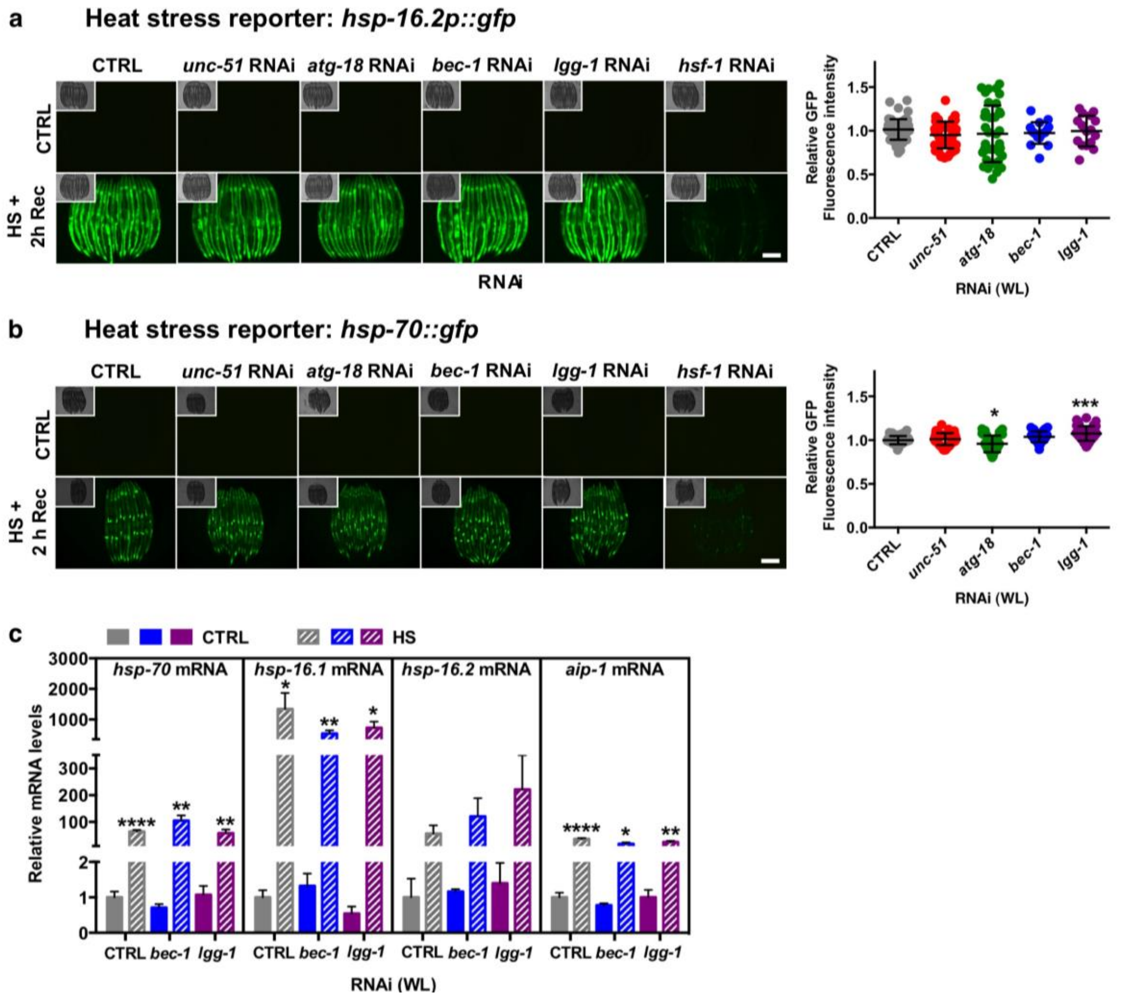

Supplementary Figure 4. Inhibition of autophagy genes does not impair the HSR. Induction of the heat shock response was measured in animals raised from hatching (WL: whole-life RNAi) on control bacteria (empty vector, CTRL) or bacteria expressing dsRNA targeting the autophagy genes *unc-51/ATG1*, *atg-18, bec-1/ATG6, lgg-1/ATG8* or *hsf-1.* (**a,b**) Images of whole animals and quantification of relative GFP fluorescence intensity of animals expressing (**a**) *hsp-16.2p::gfp* or (**b**) *hsp-70p::gfp* on day 1 of adulthood. Animals were maintained under control conditions (CTRL) or subjected to 1 h of heat shock at 36°C (HS) followed by 2 h recovery. Scale bar: 200 μm. Data are displayed as the relative fluorescence intensity of after HS compared with CTRL animals and are the mean ± s.e.m. of N = 16-59 animals pooled from 2-5 independent experiments. *$P < 0.05$, ***$P < 0.001$ by one-way ANOVA. (**c**) Transcript levels of genes involved in the heat shock response in wild-type (N2) animals maintained under control conditions (CTRL) or subjected to heat shock for 1 h at 36°C (HS). Data are the mean ± s.e.m. of three biological replicates, each with three technical replicates, and are normalized to the mean expression levels of two housekeeping genes. Error bars indicate s.e.m. No symbol: $P > 0.05$, *$P < 0.05$, **$P < 0.01$, ****$P < 0.0001$ by multiple *t*-test.

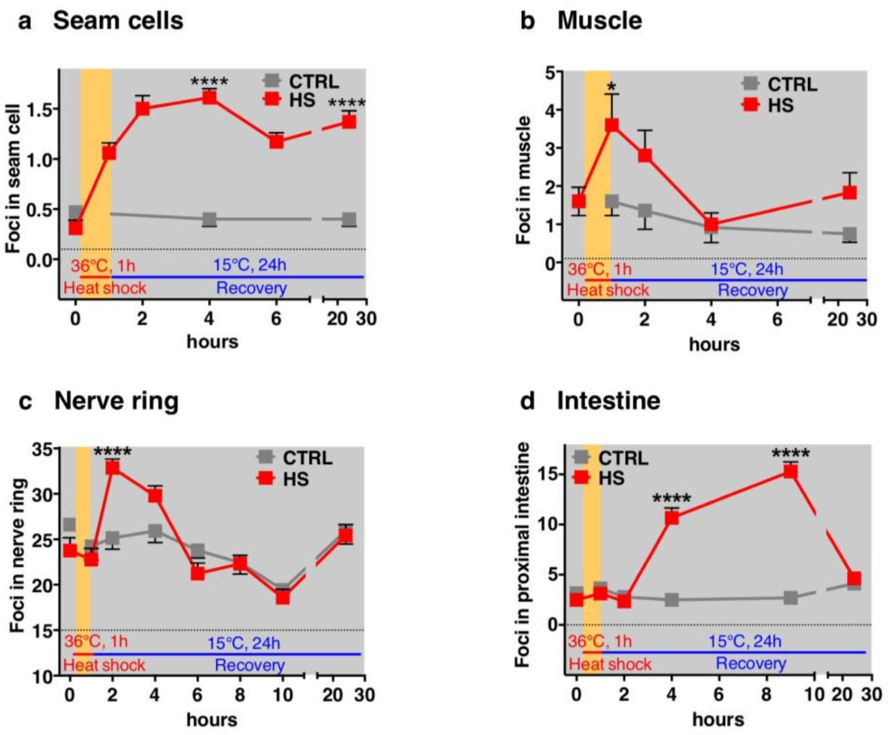

Supplementary Figure 5. Hormetic heat shock induces autophagy with distinct, tissue-specific kinetics. GFP::LGG-1/Atg8 punctae were counted on day 1 of adulthood in *C. elegans* expressing (**a,b,d**) *lgg-1p::gfp::lgg-1* or (**c**) *rgef-1p::gfp::lgg-1* maintained under control conditions (CTRL, solid bars) or subjected to heat shock for 1 h at 36°C (HS, striped bars) followed by a recovery period. Punctae were examined in (**a**) hypodermal seam cells (N = 64-116 cells), (**b**) body-wall muscle (N = 10-12 animals), (**c**) nerve ring neurons (N = 12-14 animals), and (**d**) intestinal cells (N = 14-17 animals). Error bars indicate s.e.m. *$P < 0.05$, ****$P < 0.0001$ by two-way ANOVA (time-course for muscle, nerve ring, and intestine) and Student's *t*-test (single time points in hypodermal seam cells). See also Supplementary Table 3 for a summary of additional experiments.

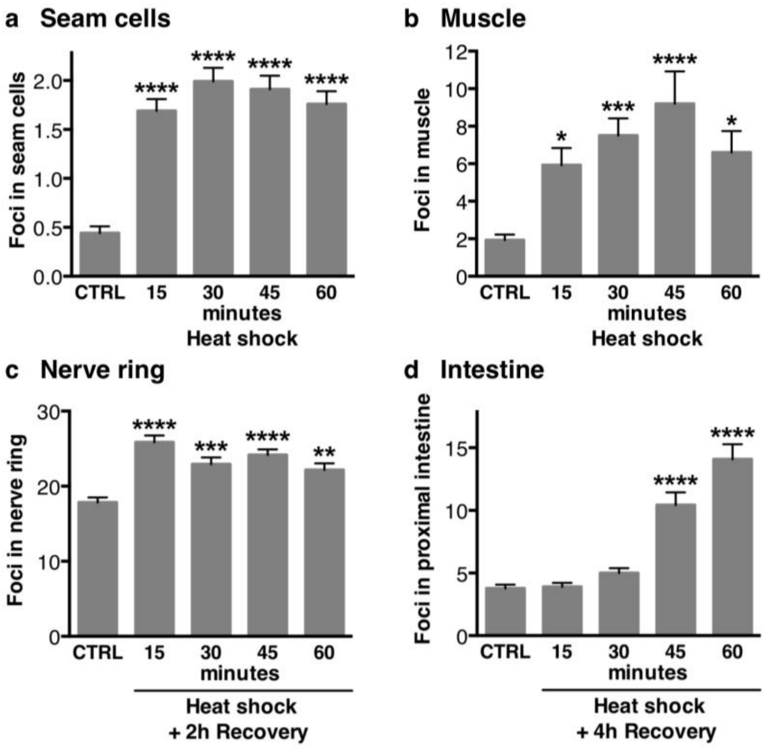

Supplementary Figure 6. The duration of hormetic heat shock differentially affects tissue-specific induction of autophagy. GFP::LGG-1/Atg8 punctae were counted on day 1 of adulthood in *C. elegans* expressing (**a,b,d**) *lgg-1p::gfp::lgg-1* or (**c**) *rgef-1p::gfp::lgg-1* maintained under control conditions (CTRL, solid bars) or subjected to 15, 30, 45, or 60 min of heat shock at 36°C followed by the indicated recovery period. Punctae were examined at the indicated recovery times in (**a**) hypodermal seam cells (N = 75-96 cells), (**b**) body-wall muscle (N = 10-12 animals), (**c**) nerve ring neurons (N = 12–13 animals), and (**d**) intestinal cells (N = 11-13 animals). Error bars indicate s.e.m. *P < 0.05, **P < 0.01, *** P < 0.001, ****P < 0.0001 by one-way ANOVA. See also Supplementary Table 6 for a summary of additional experiments.

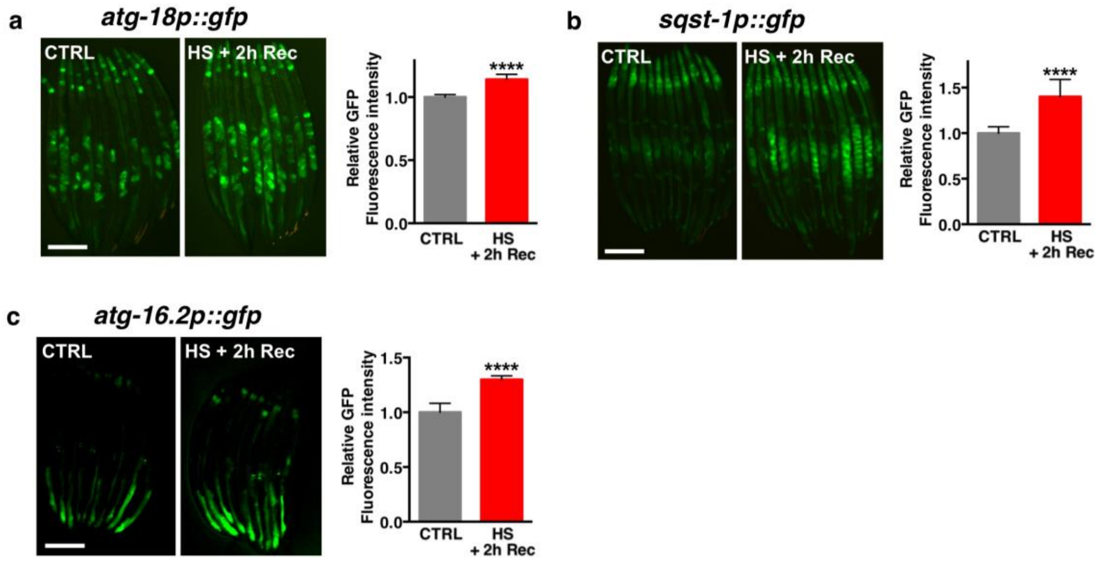

Supplementary Figure 7. Hormetic heat shock induces transcriptional autophagy reporters. Relative GFP fluorescence intensity was assessed on day 1 of adulthood in animals expressing (**a**) *atg-18p::gfp,* (**b**) *sqst-1p::gfp*, and (**c**) *atg-16.2p::gfp* and maintained under control conditions (CTRL) or subjected to 1 h of heat shock 36°C (HS) followed by 2 h recovery (Rec). Scale bar: 200 µm. Error bars indicate s.e.m. of N=10-13 animals. *P < 0.05, ****P < 0.0001 by Student's t-test. The experiments were performed at least three times with similar results.

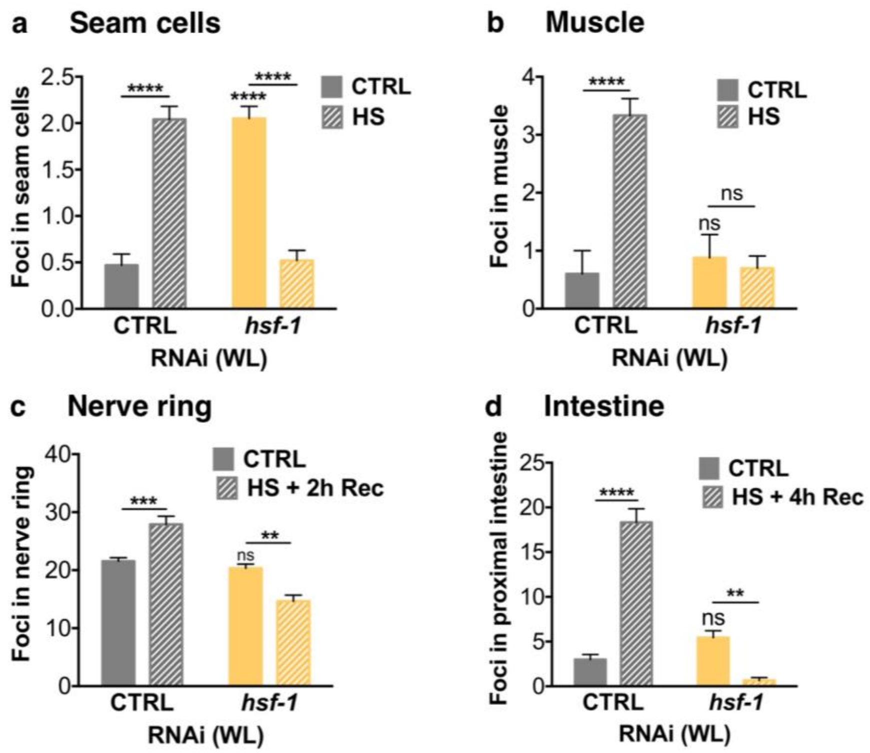

Supplementary Figure 8. Reduction of *hsf-1* limits autophagy induction in most tissues. GFP::LGG-1/Atg8 punctae were counted on day 1 of adulthood in *C. elegans* expressing (**a,b,d**) *lgg-1p::gfp::lgg-1* or (**c**) *rgef-1p::gfp::lgg-1* maintained under control conditions (CTRL, solid bars) or subjected to heat shock for 1 h at 36°C (HS, striped bars) followed by the indicated recovery period (Rec). Animals were raised from hatching (WL: whole-life RNAi) on control bacteria (empty vector, CTRL) or bacteria expressing dsRNA targeting *hsf-1.* Punctae were examined in (**a**) hypodermal seam cells (N = 47-57 cells), (**b**) body-wall muscle (N = 8-10 animals), (**c**) nerve ring neurons (N = 11-12 animals), and (**d**) intestinal cells (N = 12-13 animals). Hypodermal seam cells were distinct from the other tissues, since *hsf-1* reduction increased the number of GFP::LGG-1/Atg8–positive punctae under control conditions in this tissue. This could be due to a block or an induction of autophagy in these specialized cells. Further experiments (e.g., flux assays with bafilomycin A) will be necessary to fully characterize autophagy activity in individual tissues in response to *hsf-1* RNAi and hormetic heat shock. Error bars indicate s.e.m. ns: $P > 0.05$, $^{*}P < 0.05$, $^{****}P < 0.0001$ by two-way ANOVA. See also Supplementary Table 5 for a summary of repeat experiments.

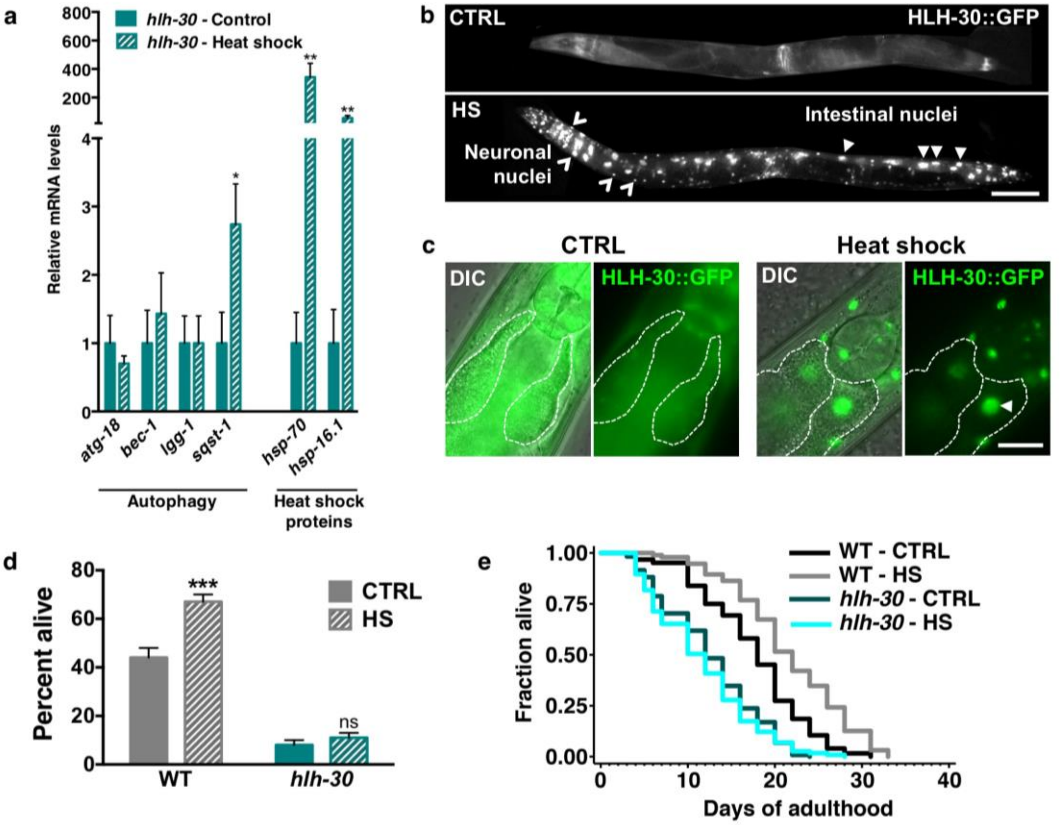

Supplementary Figure 9. Heat shock engages the helix-loop-helix transcription factor HLH-30. (**a**) Transcript levels of autophagy genes in *hlh-30(tm1978)* mutants maintained under control conditions or subjected to heat shock for 1 h at 36°C. Data are the mean ± s.e.m. of three biological replicates, each with three technical replicates, and are normalized to the mean expression levels of two housekeeping genes. Error bars indicate s.e.m. **P* < 0.05, ***P* < 0.01, no symbol: *P* > 0.05 by multiple *t*-tests. (**b,c**) Nuclear localization of HLH-30::GFP in (**b**) whole animals and (**c**) proximal intestinal cells and terminal pharyngeal bulb visualized by fluorescence microscopy on day 1 of adulthood. Animals expressed HLH-30::GFP and were maintained under control conditions (CTRL) or subjected to heat shock for 1 h at 36°C (HS). Outlines show proximal intestinal cells, closed arrowheads point to intestinal nuclei, and open arrowheads point to neuronal nuclei. Scale bar: 20 μm. (**d**) Survival of wild-type (WT, N2) animals and *hlh-30(tm1978)* mutants maintained under control conditions (CTRL, solid bars) or subjected to heat shock for 30 min at 36°C (HS, striped bars) on day 1 of adulthood followed by incubation for 8 h at 36°C on day 5 of adulthood (N = 93–112 animals, *n* = 4 plates). See Supplementary Table 9 for details of repeat experiments. Error bars indicate s.e.m. ns: *P* > 0.05, ****P* < 0.001, *****P* < 0.0001 by two-way ANOVA. (**e**) Lifespan analysis of WT (N2) animals and *hlh-30(tm1978)* mutants maintained under control conditions (CTRL) or subjected to heat shock for 1 h at 36°C (HS) on day 1 of adulthood. WT-CTRL animals (17.6 days) compared to WT-HS animals (21.7 days): *P*<0.0001, *hlh-30(tm1978)*-CTRL animals (12.5 days) compared to *hlh-30(tm1978)*-HS (11.6 days): *P*=0.3. See Supplementary Table 10 for details of lifespan analyses and replicate experiments.

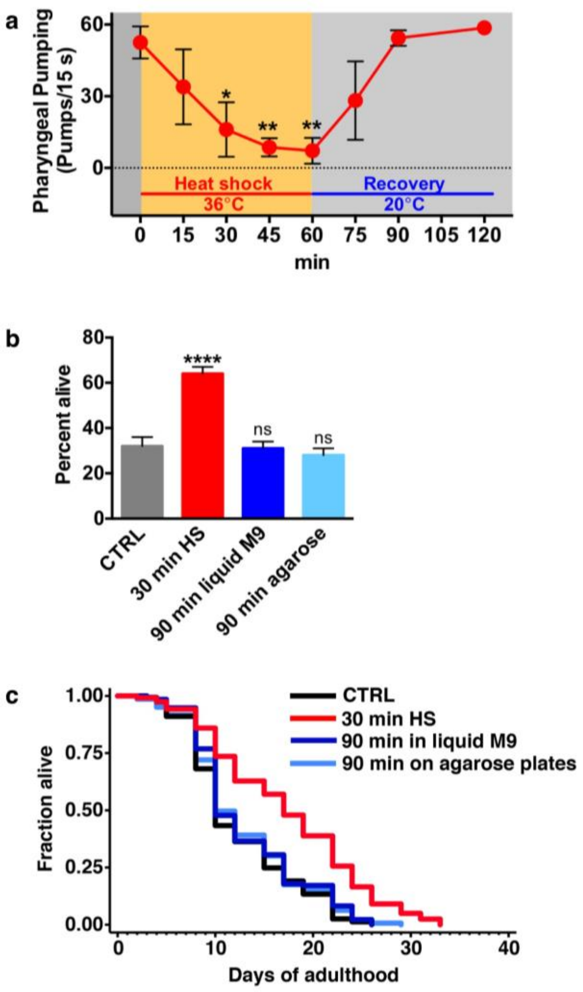

Supplementary Figure 10. Short-term decrease in food intake does not affect stress resistance or lifespan. (**a**) Pharyngeal pumping of wild-type (WT, N2) animals was quantified on day 1 of adulthood before and after heat shock at 36°C and subsequent recovery at 20°C (N = 12–16 animals per time point). Error bars indicate s.d. *P < 0.05, **P < 0.01, no symbol: P > 0.05 by one-way ANOVA compared to 0 min time point. This experiment was repeated three times with similar results. (**b**) Survival of WT (N2) animals maintained under control conditions (CTRL), subjected to heat shock for 1 h at 36°C (HS), or subjected to 90 min of food deprivation by incubating animals in liquid M9 medium or for 90 min on agarose plates without food on day 1 of adulthood and then incubated for 7 h at 36°C on day 3 of adulthood (N = 86-89 animals, *n* = 4 plates). Error bars indicate s.e.m. ns: *P* > 0.05, ****P < 0.0001 by one-way ANOVA. See Supplementary Table 1 for details of replicate experiments. (c) Lifespan analysis of WT (N2) animals maintained under control conditions (CTRL) or subjected to heat shock for 1 h at 36°C (HS), or subjected to 90 min of food deprivation by incubating animals in liquid M9 media or on agarose plates without food on day 1 of adulthood. . WT-CTRL animals (12.4 days, N = 157) compared to WT-HS animals (17.4 days, N = 131): *P*<0.0001, compared to WT-90 min in liquid M9 (13.1 days, N = 134) *P*=0.2, compared to WT-90 min on agarose plates (12.9 days, N = 143) *P*=0.3. See Supplementary Table 2 for details of lifespan analyses and replicate experiments.

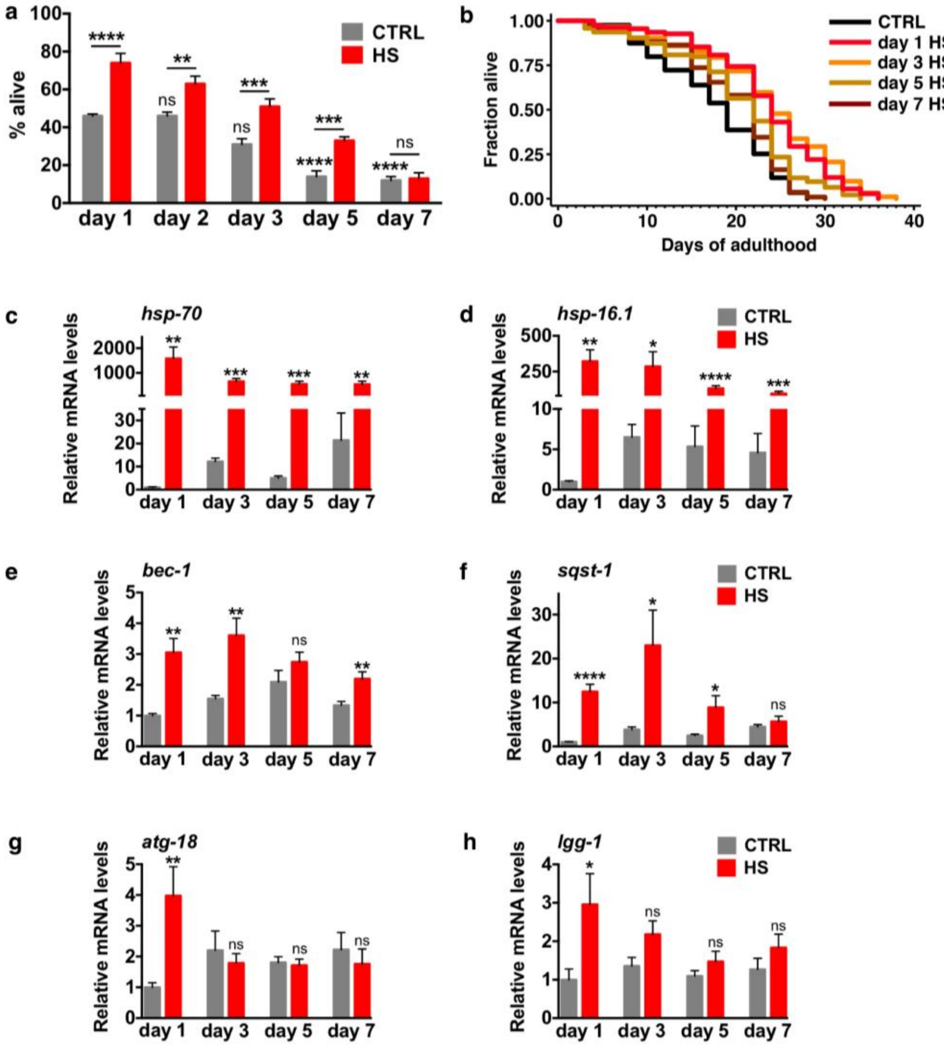

Supplementary Figure 11. Effects of hormetic heat shock in *C. elegans* at different ages. (**a**) Survival of wild-type (WT, N2) animals maintained under control conditions (CTRL) or subjected to heat shock for 1 h at 36°C (HS) on day 1, 2, 3, 5, or 7 of adulthood followed by incubation for 7 h at 36°C two days later (N = 75–133 animals, *n* = 4 plates). Error bars indicate s.e.m. ***P* < 0.01, ****P* < 0.001, *****P* < 0.0001 by two-way ANOVA. See Supplementary Table 1 for details of replicate experiments. (**b**) Lifespan analysis of WT animals maintained under control conditions (CTRL) or subjected to heat shock for 1 h at 36°C (HS) on day 1, 3, 5, or 7 of adulthood. WT-CTRL animals (17.7 days, N = 119) compared to WT-HS, d1 animals (23.4 days, N = 109): *P* < 0.0001, compared to WT-HS d3 (23.6 days, N = 92) *P* < 0.0001, compared to WT-HS d5 (20.3 days, N=94), compared to WT-HS d7 (19.6 days, N = 110) *P* = 0.04. See Supplementary Table 2 for details of lifespan analyses and replicate experiments. (**c–h**) Transcript levels of (**c**) *hsp-70*, (**d**) *hsp-16.1*, (**e**) *bec-1/ATG6*, (**f**) *sqst-1*, (**g**) *atg-18*, and (**h**) *lgg-1/ATG8* in WT animals maintained under control conditions (CTRL) or subjected to heat shock for 1 h at 36°C (HS) on day 1, 3, 5, or 7 of adulthood. Data are the mean ± s.e.m. of three biological replicates, each with three technical replicates, and are normalized to the mean expression levels of two housekeeping genes. ns: *P* > 0.05, **P* < 0.05, ***P* < 0.01, ****P* < 0.001, *****P* < 0.0001 by multiple *t*-tests.

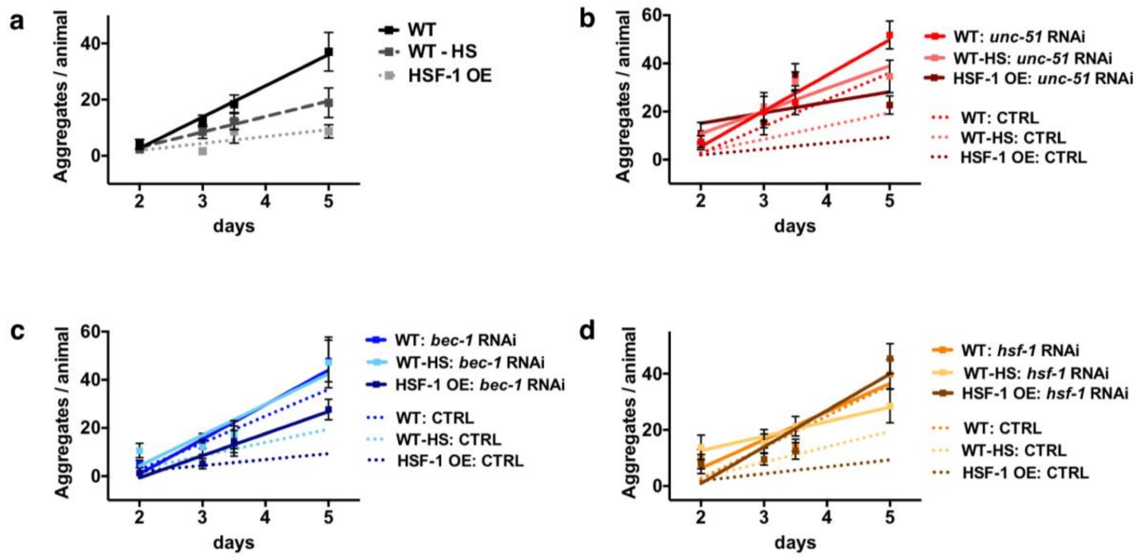

Supplementary Figure 12. Reduction of autophagy gene expression exacerbates intestinal PolyQ aggregation. PolyQ aggregates were counted in wild-type (WT) or HSF-1–overexpressing (HSF-1 OE) animals expressing intestinal PolyQ44 protein (*vha-6p::Q44::yfp*). Animals were maintained under control conditions or subjected to 1 h of heat shock at 36 °C (HS) on day 1 of adulthood. (**a**) Animals fed control bacteria. (**b–d**) Animals raised from hatching on control bacteria expressing empty vector (CTRL; dotted lines) or bacteria expressing dsRNA targeting the autophagy genes (**b**) *unc-51/ATG1*, (**c**) *bec-1/ATG6,* or (**d**) *hsf-1* (solid lines). The number of intestinal PolyQ aggregates in 10–30 animals was counted on days 2–5 and the data were plotted using linear regression. Error bars indicate s.e.m. The experiment was repeated at least three times with similar results.

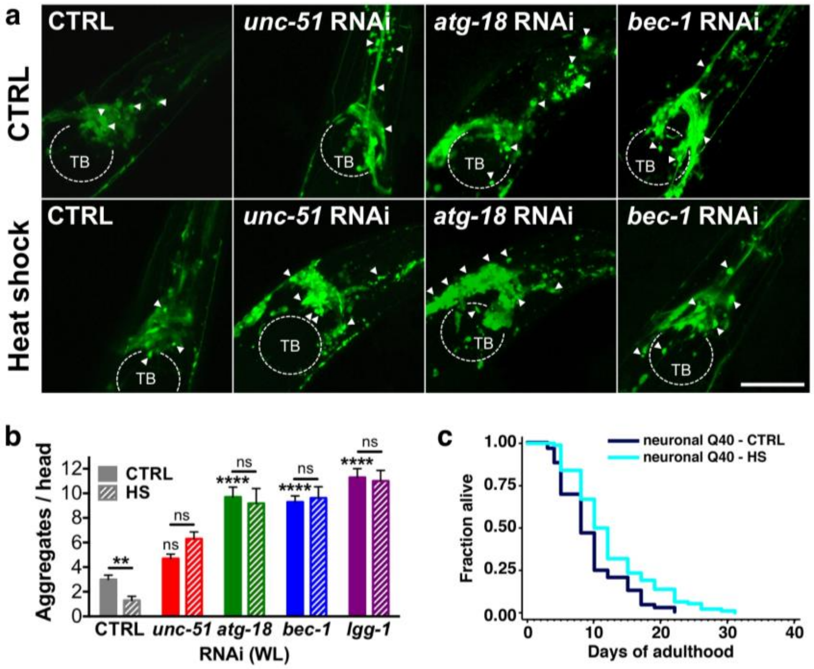

Supplementary Figure 13. Hormetic heat shock reduces neuronal PolyQ protein aggregation in an autophagy-dependent manner. (**a**) Neuronal PolyQ aggregates were counted on day 7 of adulthood in animals maintained under control conditions (CTRL) or subjected to 1 h of heat shock at 36°C (HS) on day 1 of adulthood. Animals expressing PolyQ40::YFP under the control of the neuronal-specific promoter *rgef-1* were fed from hatching with control bacteria (empty vector) or bacteria expressing dsRNA targeting *unc-51/ATG1*, *atg-18*, or *bec-1/ATG6.* Arrowheads indicate prominent aggregates. TB: terminal pharyngeal bulb. Scale bar: 20 μm. (**b**) Quantification of neuronal PolyQ aggregates on day 7 of adulthood in animals fed from hatching (whole-life RNAi, WL) with control bacteria (empty vector, CTRL) or bacteria expressing dsRNA targeting *unc-51/ATG1*, *atg-18*, *bec-1/ATG6,* or *lgg-1/ATG8* (N=11–15). Animals fed bacteria expressing dsRNA targeting *hsf-1* did not survive until day 7 of adulthood (data not shown). Error bars indicate s.e.m. ns: $P > 0.05$, $^{**}P < 0.01$, $^{****}P < 0.0001$ by two-way ANOVA. The experiments were repeated at least three times with similar results. (**c**) Lifespan analysis of animals expressing neuronal PolyQ40::YFP and subjected to hormetic heat shock (HS) on day 1 of adulthood. Neuronal Q40-CTRL animals (9.4 days, N = 118) compared to Neuronal Q40-HS animals (12.4 days, N = 94): $P = 0.0001$. See Supplementary Table 13 for details of lifespan analyses and replicate experiments.

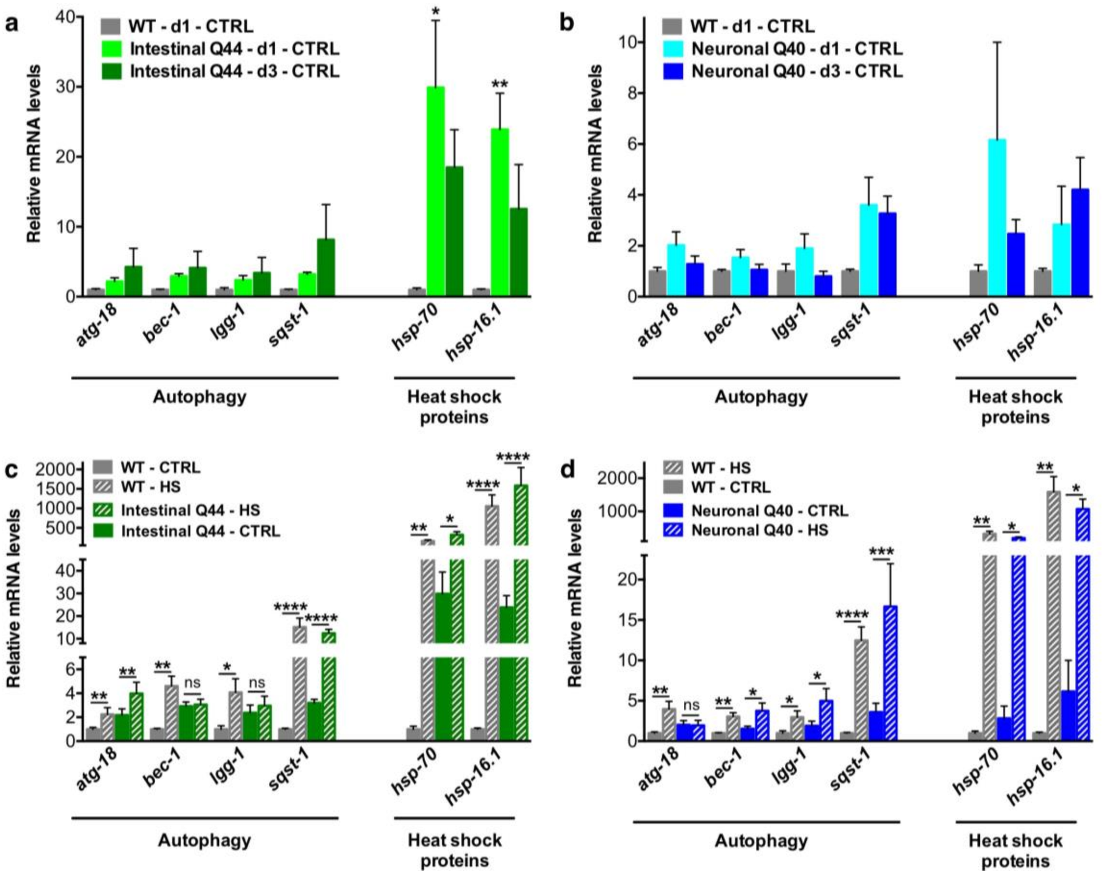

Supplementary Figure 14. Hormetic heat shock induces autophagy gene transcription in animals expressing PolyQ proteins. (**a,b**) Transcript levels of autophagy genes were measured in wild-type (WT, N2) animals on day 1 of adulthood and in animals expressing (**a**) intestinal PolyQ44 and (**b**) neuronal PolyQ40::YFP on day 1 and 3 of adulthood. (**c,d**) Transcript levels of autophagy genes were measured in WT animals and animals expressing Q44::YFP in the intestine (**c**) or Q40::YFP neurons (**d**) and maintained under control conditions (CTRL) or subjected to heat shock for 1 h at 36°C (HS) on day 1 of adulthood. Data are the mean ± s.e.m. of three biological replicates, each with three technical replicates, and are normalized to the mean expression levels of two housekeeping genes. No symbol or ns: $P > 0.05$, *$P < 0.05$, **$P < 0.01$, ***$P < 0.001$, ****$P < 0.0001$ by one-way ANOVA.

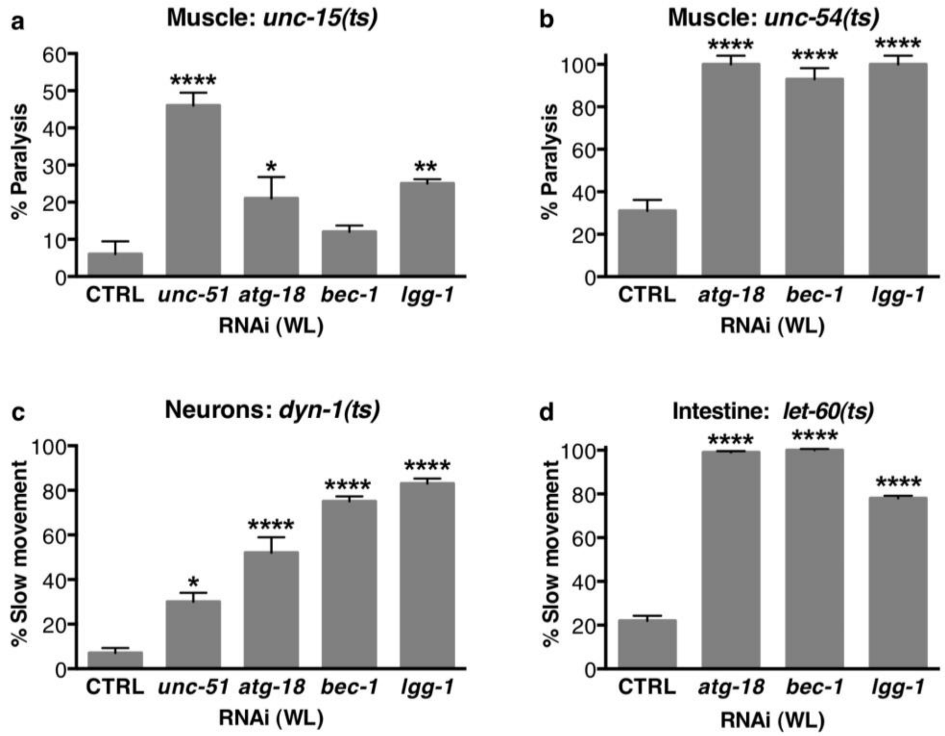

Supplementary Figure 15. Reduction of autophagy genes exacerbates movement defects in temperature-sensitive protein misfolding mutants. Paralysis was assessed in animals carrying temperature-sensitive (ts) missense mutations in (**a**) *unc-15(ts)* (paramyosin), (**b**) *unc-54(ts)* (myosin), both of which affect muscle function, (**c**) *dyn-1(ts)* (dynamin GTPase), which affects neuronal function, and (**d**) *let-60(ts)* (Ras protein), which affects intestinal and hypodermal cell function. Animals were fed from hatching (whole-life RNAi, WL) with control bacteria (empty vector, CTRL) or bacteria expressing dsRNA targeting the indicated autophagy genes. On day 5 of adulthood, slow movement or paralysis was scored at the semi-permissive temperature of 20°C (N = 10–30 animals, n = 3 plates). The experiment was performed at least three times with similar results. Error bars indicate s.e.m. *$P < 0.05$, **$P < 0.01$, ***$P < 0.001$, ****$P < 0.0001$ by one-way ANOVA.

2. Supplementary Tables

Supplementary Table 1: Thermorecovery analyses of wild-type animals after hormetic heat shock.

Exp	RNAi	Heat shock conditions	Control			Heat shock and 0-hour Recovery			*P* value (CTRL vs HS)
			% alive ± s.e.m.	N	*P* value (vs CTRL)	% alive ± s.e.m.	N	*P* value (vs CTRL)	
1	CTRL	HS: 1 h, 36°C, d1 TR: d5, 6 h	46 ± 7	121	--	75 ± 2	111	--	*
	unc-51		52 ± 4	80	ns	65 ± 4	92	ns	ns
	atg-18		54 ± 4	108	ns	70 ± 8	82	ns	ns
	bec-1		53 ± 3	97	ns	62 ± 7	109	ns	ns
	lgg-1		57 ± 2	118	ns	59 ± 6	117	ns	ns
2[1]	CTRL	HS: 30 min, 36°C, d1 TR: d4, 9 h	25 ± 3	65	--	62 ± 3	90	--	***
	unc-51					19 ± 3	89	****	ns
	bec-1					26 ± 6	73	****	ns
	lgg-1					25 ± 3	79	****	ns
3	CTRL	HS: 30 min, 36°C, d1 TR: d4, 9 h	10 ± 2	105	--	55 ± 7	94	--	***
	unc-51					18 ± 3	87	***	ns
	bec-1					11 ± 2	106	***	ns
	lgg-1					24 ± 8	101	**	ns
4	CTRL	HS: 30 min, 36°C, d1 TR: d4, 8 h	13 ± 2	86	--	41 ± 3	105	--	***
	unc-51					14 ± 3	100	***	ns
	lgg-1					15 ± 1	84	***	ns
	lmp-1					7 ± 3	86	***	ns
5	CTRL	HS: 30 min, 36°C, d1 TR: d5, 8 h	26 ± 1	111	--	47 ± 2	90	--	***
	unc-51					25 ± 2	107	****	ns
	bec-1					24 ± 2	106	****	ns
	lgg-1					15 ± 2	90	****	ns
6	CTRL	HS: 30 min, 36°C, d1 TR: d4, 8.5 h	4 ± 2	87	--	14±4	101	--	*
	unc-51					2±1	87	**	ns
	bec-1					3±1	90	*	ns
	lgg-1					4±3	90	*	ns
	lmp-1					0±0	86	**	ns
	hsf-1					1±1	89	**	ns
7	CTRL	HS: 30 min, 36°C, d1 TR: d4, 7 h	23±3	101	--	40 ± 6	112	--	*
	bec-1					20 ± 4	67	*	ns
	lgg-1					7 ± 2	93	**	*
8	CTRL	HS: 1 h, 36°C, d1 TR: d4, 7 h	40 ± 3	110	--	69 ± 5	92	--	***
	bec-1		35 ± 4	95	ns	27 ± 4	65	***	ns
9	CTRL	HS: 30 min, 36°C, d1 TR: d3, 7 h	26 ± 2	105	--	60±7	102	--	****
	CTRL	HS: 30 min, 36°C, d3 TR: d5, 7 h	45 ± 5	94	ns	67 ± 7	91	ns	**
	CTRL	HS: 30 min, 36°C, d5 TR: d7, 7 h	30 ± 2	89	ns	42 ± 5	74	ns	ns
	CTRL	HS: 30 min, 36°C, d7 TR: d9, 7 h	20 ± 2	99	ns	30 ± 3	90	**	ns

(Supplementary Table 1 continued)

Exp	RNAi	Heat shock conditions	Control			Heat shock and 0-hour Recovery			*P* value (CTRL vs HS)
			% alive ± s.e.m.	N	*P* value	% alive ± s.e.m.	N	*P* value	
10	CTRL	HS: 30 min, 36°C, d1<br>TR: d3, 7 h	25 ± 1	153	--	56 ± 3	126	--	****
	CTRL	HS: 30 min, 36°C, d3<br>TR: d5, 7 h	33 ± 3	133	ns	55 ± 4	115	ns	**
	CTRL	HS: 30 min, 36°C, d5<br>TR: d7, 7 h	22 ± 2	93	ns	33 ± 4	85	ns	ns
	CTRL	HS: 30 min, 36°C, d7<br>TR: d9, 7 h	21 ± 5	51	ns	17 ± 1	60	**	ns
11[2]	CTRL	HS: 30 min, 36°C, d1<br>TR: d3, 7 h	46 ± 1	125	--	74 ± 5	115	--	****
	CTRL	HS: 30 min, 36°C, d2<br>TR: d4, 7 h	46 ± 2	133	ns	63 ± 4	92	ns	**
	CTRL	HS: 30 min, 36°C, d3<br>TR: d5, 7 h	31 ± 3	107	ns	51 ± 4	102	***	***
	CTRL	HS: 30 min, 36°C, d5<br>TR: d7, 7 h	14 ± 3	89	****	33 ± 2	86	****	***
	CTRL	HS: 30 min, 36°C, d7<br>TR: d9, 7 h	12 ± 2	79	****	13 ± 3	75	****	ns
12[3]	CTRL	HS: 30 min, 36°C, d1<br>TR: d3, 7 h	32 ± 4	86	--	64 ± 3	89	--	****
	CTRL	Starvation: 90 min, M9, d1<br>TR: d3, 7 h	31 ± 3	87	ns				
	CTRL	Starvation: 90 min, agarose,d1<br>TR: d3, 7 h	28 ± 3	87	ns				
13	CTRL	HS: 30 min, 36°C, d1<br>TR: d3, 8.5 h	7 ± 2	111	--	21 ± 2	109	--	**
	CTRL	Starvation: 90 min, M9, d1<br>TR: d3, 8.5 h	8 ± 2	104	ns				
	CTRL	Starvation: 90 min, agarose,d1<br>TR: d3, 8.5 h	10 ± 3	110	ns				
14	CTRL	HS: 30 min, 36°C, d1<br>TR: d3, 7 h	88 ± 2	117	--	98 ± 1	106	--	*
	CTRL	Starvation: 90 min, M9, d1<br>TR: d3, 8 h	84 ± 3	113	ns				
	CTRL	Starvation: 90 min, agarose,d1<br>TR: d3, 8 h	88 ± 3	132	ns				

Supplementary Table 1: Survival analysis of wild-type animals fed from day 1 of adulthood with control bacteria (empty vector, CTRL) or bacteria expressing dsRNA targeting the indicated genes. Animals were maintained under control conditions or subjected to 30–60 min of hormetic heat shock (HS: 36°C) on the indicated day of adulthood or to food deprivation as indicated (experiments 12–14). Thermorecovery (TR) was tested on the indicated days of adulthood by incubating animals at 36°C for 6–9 h followed by ~20 h of recovery. Exp: experiment number (in chronological order); s.e.m.: standard error of the mean; N: number of animals analyzed (see Methods). The mean ± s.e.m. percentage survival of experimental and control groups were analyzed using Student's *t*-test, one-way ANOVA, or two-way ANOVA. ns: $P > 0.05$, *$P < 0.05$, **$P < 0.01$, ***$P < 0.001$, ****$P < 0.0001$. [1]: data depicted in **Fig. 3a**; [2]: data depicted in **Supplementary Fig. 11a**; [3]: data depicted in **Supplementary Fig. 10b**.

Supplementary Table 2: Lifespan analyses of wild-type animals after hormetic heat shock.

Exp	RNAi	Heat shock	MLS (days)	N	% MLS	*P* value
1	CTRL	-	22.4	106/124		
	CTRL	1 h, 36°C, d1	26.3	99/129	+17.4%	0.0001 (****)
2	CTRL	-	15.4	93/120		
	CTRL	1 h, 36°C, d1	16.7	100/120	+8.4%	0.1 (ns)
3	CTRL		18.3	95/122		
	CTRL	1 h, 36°C, d1	19.9	94/121	+8.7%	0.0007 (***)
4	CTRL		17.1	106/137		
	CTRL	1 h, 36°C, d1	20.7	82/129	+21.1%	0.0005 (***)
	unc-51	1 h, 36°C, d1	15.4	106/125	-10.0%	0.1 (ns)
	atg-18	1 h, 36°C, d1	11.6	101/129	-32.2%	<0.0001 (****)
	bec-1	1 h, 36°C, d1	16.5	113/123	-3.5%	0.1 (ns)
	lgg-1	1 h, 36°C, d1	15.3	103/126	-10.5%	0.08 (ns)
5	CTRL	-	15.7	116/120		
	CTRL	1 h, 36°C, d1	17.7	83/120	+12.7%	0.01 (**)
	unc-51	1 h, 36°C, d1	15.5	110/120	-1.3%	1.0 (ns)
	atg-18	1 h, 36°C, d1	16.5	105/120	+5.10%	0.2 (ns)
	bec-1	1 h, 36°C, d1	15.5	93/120	-1.3%	0.9 (ns)
	lgg-1	1 h, 36°C, d1	15.6	102/120	-0.6%	0.8 (ns)
6[1]	CTRL	-	19.2	104/125		
	CTRL	30 min, 36°C, d1	23.7	94/111	+23.4%	<0.0001 (****)
	unc-51	-	18.5	110/122	-3.5%	0.3 (ns)
	unc-51	30 min, 36°C, d1	17.5	107/128	-8.8%	0.04 (*)
	bec-1	-	19.2	116/130	0%	0.2 (ns)
	bec-1	30 min, 36°C, d1	18.4	112/126	-4.2%	0.3 (ns)
	lgg-1	-	18.1	108/125	-5.7%	0.06 (ns)
	lgg-1	30 min, 36°C, d1	17.9	79/126	-6.8%	0.7 (ns)
7	CTRL	-	17.3	177/184		
	CTRL	30 min, 36°C, d1	22.4	140/159	+29.5%	<0.0001 (****)
	unc-51	-	17.3	137/141	0%	0.5 (ns)
	unc-51	30 min, 36°C, d1	16.5	148/154	-4.6%	0.4 (ns)
	lgg-1	-	19.3	142/146	+11.6%	0.1 (ns)
	lgg-1	30 min, 36°C, d1	17.4	171/180	-9.8%	0.2 (ns)
8	--	-	14.4	107/120		
	--	30 min, 36°C, d1	21.4	85/121	+48.6%	<0.0001 (****)
	--	30 min, 36°C, d3	16.6	103/128	+15.3%	0.006 (***)
	--	30 min, 36°C, d5	17.3	89/120	+20.1%	0.0006 (***)
	--	30 min, 36°C, d7	14.4	95/120	0%	0.7 (ns)
	--	90 min in liquid M91	15.1	94/122	+4.9%	0.2 (ns)

(Supplementary Table 2 continued)

Exp	RNAi	Heat shock	MLS (days)	N	% MLS	*P* value
9[2]	--	-	12.4	157/162		
	--	30 min, 36°C, d1	17.4	131/145	+40.3%	<0.0001(****)
	--	30 min, 36°C, d3	14.6	130/160	+17.7%	0.0006 (***)
	--	30 min, 36°C, d5	14.3	142/164	+15.3%	0.001 (***)
	--	30 min, 36°C, d7	14.5	140/160	+16.9%	0.001 (***)
	--	90 min in liquid M9	13.1	134/151	+5.6%	0.2 (ns)
	--	90 min on agarose plates	12.9	143/161	+4.0%	0.3 (ns)
10[3]	--	-	17.7	119/124		
	--	30 min, 36°C, d1	23.4	109/124	+32.2%	<0.0001(****)
	--	30 min, 36°C, d3	23.6	92/127	+33.3%	<0.0001(****)
	--	30 min, 36°C, d5	20.3	94/136	+14.7%	0.0004 (***)
	--	30 min, 36°C, d7	19.6	110/122	+10.7%	0.04 (*)
	--	90 min in liquid M9	17.1	122/127	-3.4%	0.8 (ns)
	--	90 min on agarose plates	16.9	115/125	-4.6%	0.6 (ns)

Supplementary Table 2: Lifespan analysis of wild-type animals subjected to hormetic heat shock at 36°C (HS) for 30–60 min on the indicated day of adulthood (d1: day 1, d3: day 3, d5: day 5, d7: day 7). Animals were fed from day 1 of adulthood with control bacteria (empty vector, CTRL) or bacteria expressing dsRNA targeting the indicated autophagy genes. If no RNAi is indicated, the animals were fed OP50 bacteria throughout life. Exp: experiment number (in chronological order); MLS: mean lifespan; N: observed deaths/total number of animals subjected to RNAi; % MLS: percentage change in lifespan compared with control; ns: $P > 0.05$, $*P < 0.05$, $**P < 0.01$, $***P < 0.001$, $****P < 0.0001$ by log-rank test. [1]: data depicted in **Fig. 3c–e**; [2]: data for CTRL and food deprivation depicted in **Supplementary Fig. 10c**; [3]: data depicted in **Supplementary Fig. 11b**.

Supplementary Table 3: Autophagy measurements in wild-type animals after hormetic heat shock.

Hypodermal seam cells							
Exp	Age	Recovery time	Control		Heat shock		*P* value
			Mean ± s.e.m.	N	Mean ± s.e.m.	N	
1	L3	0 h	0.4 ± 0.4	189	0.9 ± 0.1	144	****
2	L3	0 h	0.3 ± 0.04	200	1.0 ± 0.2	229	****
3	L3	0 h	0.4 ± 0.06	87	1.3 ± 0.1	111	****
4	L3	0 h	0.3 ± 0.03	234	0.9 ± 0.1	190	****
5	L3	0 h	0.2 ± 0.04	169	1.0 ± 0.1	152	***
6	L3	0 h	0.3 ± 0.04	173	0.9 ± 0.1	162	****
7	D1	0 h	0.3 ± 0.06	83	0.8 ± 0.1	70	***
8	D1	0 h	0.4 ± 0.07	82	3.6 ± 0.2	66	****
10	D1	0 h	0.5 ± 0.12	49	2.0 ± 0.1	47	****
11	D1	0 h	0.2 ± 0.03	148	1.0 ± 0.1	111	****
		2 h			1.7 ± 0.1	126	****
		4 h			1.1 ± 0.1	115	****
		6 h			0.9 ± 0.1	125	****
		24 h			1.01 ± 0.1	113	****
12[2]	D1	Pre-HS	0.5 ± 0.1	101	0.3 ± 0.04	191	ns
		0 h			1.1 ± 0.1	124	****<br>(vs pre-HS)
		2 h			1.5 ± 0.1	120	****
		4 h	0.4 ± 0.1	114	1.6 ± 0.1	122	****
		6 h			1.2 ± 0.1	119	****
		24 h	0.4 ± 0.1	100	1.4 ± 0.1	87	****
13	D1	0 h	0.3 ± 0.1	120	1.2 ± 0.1	125	****
		2 h			2.0 ± 0.1	119	****
		4 h			2.0 ± 0.1	130	****
		6 h			2.0 ± 0.1	148	****
		24 h			2.1 ± 0.1	104	****
14	D1	0 h	0.3 ± 0.1	97	1.2 ± 0.1	112	****
		2 h			2.0 ± 0.1	64	****
		4 h	0.4 ± 0.1	115	1.7 ± 0.1	87	****
		6 h			1.8 ± 0.1	116	****
		24 h	0.4 ± 0.1	103	1.6 ± 0.1	105	****
15	L4	0 h	0.5 ± 0.1	113	1.0 ± 0.1	81	**
		2 h	0.4 ± 0.1	106	1.1 ± 0.1	97	****
		24 h	0.5 ± 0.1	101	0.8 ± 0.2	51	ns
16[1]	D1	0 h	0.5 ± 0.1	98	1.9 ± 0.2	63	****
		2 h			2.0 ± 0.1	98	****
		24 h			1.7 ± 0.1	83	****

(Supplementary Table 3 continued)

Intestine							
Exp	Age	Recovery time	Control		Heat shock		*P* value
			Mean ± s.e.m.	N	Mean ± s.e.m.	N	
1	D1	4 h	5.9 ± 1.0	7	11.0 ± 2.0	10	****
2	D1	4 h	6.0 ± 0.5	12	9.6 ± 1.0	12	**
3	D1	4 h	3.0 ± 0.3	20	5.0 ± 0.8	20	***
4[1]	D1	4 h	3.0 ± 1.2	16	19.3 ± 4.8	14	****
5	D1	4 h	2.3 ± 0.5	8	10.3 ± 1.5	10	****
6[3]	D1	4 h	8.0 ± 1.1	5	27.4 ± 1.0	7	****
7	D1	0 h	3.0 ± 0.3	20	0.8 ± 028	20	****
		4 h	2.1 ± 0.2	20	5.0 ± 0.8	20	***
		24 h	2.9 ± 0.3	20	2.2 ± 0.3	20	ns
		72 h	3.5 ± 0.30	20	3.4 ± 0.2	20	ns
8	D1	0 h	2.6 ± 0.4	20	2.7 ± 0.4	20	ns
		2 h	2.7 ± 0.3	20	3.3 ± 0.3	20	ns
		4 h	2.5 ± 0.3	20	7.1 ± 0.7	20	***
		6 h	2.0 ± 0.3	20	7.6 ± 0.6	20	****
		24 h	1.6 ± 0.3	20	1.7 ± 0.3	20	ns
9[2]	D1	0 h	3.6 ± 0.4	14	3.1 ± 0.5	14	ns
		2 h	2.8 ± 0.4	15	2.35 ± 0.3	17	ns
		4 h	2.5 ± 0.2	14	10.7 ± 1.0	15	****
		9 h	2.7 ± 0.3	17	15.3 ± 1.0	17	****
		24 h	4.1 ± 0.4	16	4.6 ± 0.3	14	ns
10	L4	2 h	3.6 ± 1.7	13	3.3 ± 1.5	17	ns
		4 h	3.8 ± 1.6	15	14.2 ± 6.5	13	****
		24 h	4.9m± 2.1	16	4.0 ± 1.3	15	ns
11	D1	2 h	1.9 ± 1.0	16	2.2 ± 1.1	13	ns
		4 h	3.7 ± 1.8	15	15.1 ± 5.6	14	****
		24 h	3.7 ± 1.4	40	3.1 ± 0.8	16	ns
12	D2	2 h	1.5 ± 1.0	14	1.9 ± 1.2	17	ns
		4 h	2.1 ± 1.5	15	10.6 ± 4.8	15	****
		24 h	3.8 ± 1.1	13	2.7 ± 1.5	14	ns
13	D1	4 h	4.9 ± 2.1	16	13.9 ± 7.6	13	****
		24 h	3.7 ± 1.5	15	4.5 ± 2.5	16	ns
	D2	4 h	3.7 ± 1.4	13	7.8 ± 3.4	13	**
		24 h	3.7 ± 1.3	15	3.4 ± 1.1	14	ns
14	D1	0 h	4.9 ± 0.7	12	4.7 ± 0.6	11	ns
		2 h			10.7 ± 2.5	10	ns
		4 h			24.3 ± 3.1	10	****
		6 h			23.7 ± 2.7	11	****
		8 h			14.8 ± 2.5	12	***
		10 h			11.6 ± 1.4	11	ns
		24 h			5.7 ± 0.4	10	ns

(Supplementary Table 3 continued)

Neurons							
Exp	Age	Recovery time	Control		Heat shock		*P* value
			Mean ± s.e.m.	N	Mean ± s.e.m.	N	
1	D1	2 h	26.1 ± 3.90	12	30.7 ± 4.05	12	**
2[1]	D1	2 h	23.8 ± 0.84	12	30.1 ± 0.84	12	***
3[4]	D1	2 h	41.8 ± 3.50	6	57.7 ± 1.61	6	**
4	D1	2 h	12.7 ± 4.30	12	18.5 ± 3.00	11	**
		24 h	16.2 ± 1.70	11	22.1 ± 4.30	7	**
		48 h	21.8 ± 3.70	12	26.0 ± 3.70	7	**
5[2]	L4	2 h	17.0 ± 2.04	14	16.6 ± 3.12	14	ns
		4 h	14.5 ± 2.96	13	14.5 ± 3.36	13	ns
		6 h	12.6 ± 1.83	12	12.8 ± 1.99	12	ns
		8 h	17.7 ± 3.61	13	16.9 ± 3.97	13	ns
		10 h	18.8 ± 3.67	12	17.8 ± 3.72	13	ns
		24 h	22.0 ± 3.36	13	20.5 ± 3.55	13	ns
6	D1	2 h	25.1 ± 4.62	14	32.9 ± 3.63	14	***
		4 h	25.9 ± 4.86	14	29.8 ± 4.06	14	ns
		6 h	23.8 ± 3.24	14	21.2 ± 4.21	13	ns
		8 h	22.4 ± 4.74	14	22.3 ± 3.38	13	ns
		10 h	19.4 ± 2.56	14	18.6 ± 3.48	14	ns
		24 h	25.9 ± 5.44	14	25.4 ± 4.50	14	ns
7	L4	0 h	17.6 ± 3.99	13	18.9 ± 2.32	14	ns
		2 h	15.8 ± 3.95	11	17.9 ± 3.17	13	ns
		24 h	19.2 ± 3.49	14	22.5 ± 3.89	15	ns
		48 h	24.2 ± 4.93	12	26.1 ± 5.62	12	ns
8	D1	0 h	18.2 ± 2.91	13	13.9 ± 3.43	14	ns
		2 h	14.6 ± 1.69	11	20.3 ± 3.77	13	**
		24 h	22.8 ± 4.66	13	24.9 ± 4.11	14	ns
		48 h	24.5 ± 6.19	13	25.4 ± 6.28	13	ns
9	D2	0 h	17.8 ± 2.98	12	15.3 ± 2.81	14	ns
		2 h	14.7 ± 2.42	12	18.5 ± 3.69	13	*
		24 h	24.0 ± 2.91	10	22.5 ± 5.47	13	ns
		48 h	26.9 ± 3.59	14	27.8 ± 3.46	12	ns
10	D1	2 h	19.6 ± 3.55	12	22.4 ± 4.06	12	ns
		4 h	18.1 ± 2.71	14	22.2 ± 2.93	11	**
		6 h	18.5 ± 2.91	12	21.0 ± 2.42	13	*
		24 h	21.7 ± 2.61	12	26.8 ± 1.76	12	****
11	D1	2 h	21.2 ± 0.58	12	26.8 ± 0.88	12	****
		24 h	27.3 ± 1.11	11	27.1 ± 1.70	10	ns

(Supplementary Table 3 continued)

Muscle							
Exp	Age	Recovery time	Control		Heat shock		*P* value
			Mean ± s.e.m.	N	Mean ± s.e.m.	N	
1[1]	D1	0 h	0.6 ± 0.5	12	6.4 ± 1.1	10	****
2	D1	0 h	3.9 ± 0.7	8	7.8 ± 1.2	9	*
3	D1	0 h	0.6 ± 0.4	10	3.3 ± 0.3	9	**
4[2]	D1	0 h	1.6 ± 0.4	10	3.6 ± 0.8	10	*
		2 h	1.4 ± 0.5	11	2.8 ± 0.7	11	ns
		4 h	0.9 ± 0.4	12	1.0 ± 0.3	12	ns
		24 h	0.8 ± 0.2	12	1.8 ± 0.5	12	ns
5	D1	0 h	10.9 ± 1.38	13	15.1 ± 2.1	13	*
		2 h			9.4 ± 1.6	13	ns
		4 h			8.1 ± 1.4	12	ns
		6 h			13.6 ± 2.3	11	ns
		8 h			9.4 ± 2.2	11	ns
		10 h			10.6 ± 1.3	12	ns

Supplementary Table 3: GFP::LGG-1-positive punctae were quantified in the hypodermal seam cells, intestine, and muscle of animals expressing *lgg-1p::gfp::lgg-1* (DA2123) and in the neurons of animals expressing *rgef-1p::gfp::lgg-1* (MAH242). Animals were maintained under control conditions or subjected to 1 h of hormetic heat shock followed by the indicated recovery time. ns: $P > 0.05$, *$P < 0.05$, **$P < 0.01$, ***$P < 0.001$, ****$P < 0.0001$ by Student's *t*-test (control vs heat shock conditions), one-way ANOVA (time-course experiments), or two-way ANOVA (control vs heat shock conditions at multiple time points). Exp: experiment number (listed in chronological order); s.e.m.: standard error of the mean; N: number of cells, animals, or image fields analyzed (see Methods). [1]: data depicted in **Fig. 1a–d**; [2]: data depicted in **Supplementary Fig. 5**; [3]: analyzed from images acquired by confocal microscopy; [4]: analyzed from Z-stack images acquired by confocal microscopy.

Supplementary Table 4: Autophagy measurements in animals expressing GFP::LGG-1(G116A) after hormetic heat shock.

Hypodermal seam cells (0-hour Recovery)										
Exp	WT					GFP::LGG-1(G116A)				
	Control		Heat shock		*P* value	Control		Heat shock		*P* value
	Mean ± s.e.m.	N	Mean ± s.e.m.	N		Mean ± s.e.m.	N	Mean ± s.e.m.	N	
1	0.36 ± 0.06	89	3.4 ± 0.2	91	****	0.02 ± 0.02	89	0.02 ± 0.1	89	ns
2[1]	0.36 ± 0.09	45	2.7 ± 0.3	51	****	0.07 ± 0.03	56	0.2 ± 0.1	60	ns
3	0.25 ±0.05	85	2.4 ± 0.1	91	****	0.03 ± 0.02	96	0.01 ± 0.01	89	ns
4	0.34 ± 0.08	79	2.7 ± 0.2	80	****	0.03 ± 0.02	94	0.04 ± 0.2	115	ns
**Intestine (4-hour Recovery)**										
Exp	WT					GFP::LGG-1(G116A)				
	Control		Heat shock		*P* value	Control		Heat shock		*P* value
	Mean ± s.e.m.	N	Mean ± s.e.m.	N		Mean ± s.e.m.	N	Mean ± s.e.m.	N	
1[1]	2.1 ± 0.4	13	9.5 ± 1.0	13	****	0 ± 0	14	0 ± 0	13	ns
2	4.7 ± 0.8	7	14.4 ± 2.2	8	****	0 ± 0	6	0 ± 0	7	ns
3	1.4 ± 0.3	10	5.5 ± 1.1	8	****	0 ± 0	11	0 ± 0	10	ns
**Muscle (0-hour Recovery)**										
Exp	WT					GFP::LGG-1(G116A)				
	Control		Heat shock		*P* value	Control		Heat shock		*P* value
	Mean ± s.e.m.	N	Mean ± s.e.m.	N		Mean ± s.e.m.	N	Mean ± s.e.m.	N	
1	0.8 ± 0.4	11	2.9 ± 0.6	12	**	0 ± 0	9	0 ± 0	11	ns
2	0.67 ± 0.2	12	2.2 ± 0.3	12	****	0 ± 0	12	0 ± 0	12	ns
3[1]	1.5 ± 0.4	11	5.2 ± 0.9	10	****	0 ± 0	11	0 ± 0	11	ns

Supplementary Table 4: GFP::LGG-1-positive punctae were quantified in the hypodermal seam cells, intestine, and muscle of animals expressing *lgg-1p::gfp::lgg-1* (DA2123) or *lgg-1p::gfp::lgg-1(G116A)* (RD202). Animals were maintained under control conditions or subjected to 1 h of hormetic heat shock followed by the indicated recovery time. ns: $P > 0.05$, **$P < 0.01$, ****$P < 0.0001$ by two-way ANOVA; Exp: experiment number (in chronological order); s.e.m.: standard error of the mean; N: number of cells or animals. [1]: data depicted in **Supplementary Fig. 2.**

Supplementary Table 5: Autophagy measurements in wild-type animals subjected to RNAi treatments and a hormetic heat shock.

Hypodermal seam cells									
Exp	Age	RNAi	Control			Heat shock and 0-hour Recovery			*P* value (CTRL vs HS)
			Mean ± s.e.m.	N	*P* value (vs CTRL)	Mean ± s.e.m.	N	*P* value (vs CTRL)	
1	L3	CTRL	0.3 ± 0.1	118	--	1.5 ± 0.1	139	--	****
	L3	*atg-13*	1.1 ± 0.1	97	****	3.3 ± 0.2	92	****	****
	L3	*atg-18*	0.3 ± 0.1	142	ns	0.6 ± 0.1	109	****	ns
2	D1	CTRL	0.3 ± 0.1	96	--	0.9 ± 0.09	158	--	***
	D1	*atg-13*	1.6 ± 0.1	91	****	1.9 ± 0.1	103	****	ns
	D1	*atg-18*	0.2 ± 0.04	150	ns	0.2 ± 0.04	139	****	ns
3	D1	CTRL	0.6 ± 0.1	105	--	15 ± 0.1	96	--	****
	D1	*unc-51*	0.6 ± 0.1	99	ns	1.8 ± 0.1	110	ns	****
	D1	*atg-13*	1.5 ± 0.1	86	****	1.8 ± 0.1	82	ns	ns
	D1	*atg-18*	0.3 ± 0.1	76	ns	0.4 ± 0.1	122	****	ns
	D1	*bec-1*	0.6 ± 0.1	121	ns	0.8 ± 0.1	105	**	ns
4	D1	CTRL	1.1 ± 0.1	77	--	1.9 ± 0.1	72	--	***
	D1	*unc-51*	1.0 ± 0.1	92	ns	1.4 ± 0.1	71	ns	ns
	D1	*atg-13*	3.1 ± 0.3	69	****	2.5 ± 0.1	85	ns	*
	D1	*atg-18*	0.4 ± 0.1	81	*	0.2 ± 0.1	103	****	ns
	D1	*bec-1*	1.9 ± 0.2	76	***	1.0 ± 0.1	76	***	****
5[1]	D1	CTRL	0.3 ± 0.1	80	--	1.5 ± 0.2	66	--	****
	D1	*unc-51*	0.2 ± 0.04	100	ns	0.1 ± 0.1	77	****	ns
	D1	*atg-18*	0.1 ± 0.04	86	ns	0.1 ± 0.04	73	****	ns
	D1	*bec-1*	0.5 ± 0.1	82	ns	0.2 ± 0.1	64	****	*
6	D1	CTRL	0.2 ± 0.04	169	--	1.0 ± 0.1	152	--	****
	D1	*hsf-1*	1.2 ± 0.1	131	****	0.7 ± 0.1	170	*	****
7	D1	CTRL	0.3 ± 0.04	173	--	0.9 ± 0.1	162		****
	D1	*hsf-1*	1.2 ± 0.1	151	****	0.7 ± 0.1	135	ns	****
8[2]	D1	CTRL	0.5 ± 0.1	49	--	2.0 ± 0.1	47	--	****
	D1	*hsf-1*	2.1 ± 0.1	57	****	0.5 ± 0.1	50	****	****
**Intestine**									
Exp	Age	RNAi	Control			Heat shock and 4-hour Recovery			*P* value (CTRL vs HS)
			Mean ± s.e.m.	N	*P* value (vs CTRL)	Mean ± s.e.m.	N	*P* value (vs CTRL)	
1	D1	CTRL	2.9 ± 0.3	17	--	4.7 ± 0.3	17	--	*
		unc-51	2.7 ± 0.4	18	ns	5.0 ± 0.6	18	ns	**
		atg-18	3.8 ± 0.5	16	ns	4.5 ± 0.5	17	ns	ns
		bec-1	5.3 ± 0.6	17	**	3.5 ± 0.6	17	ns	*

(Supplementary Table 5 continued)

Exp	Age	RNAi	Control			Heat shock and 4-hour Recovery			*P* value (CTRL vs HS)
			Mean ± s.e.m.	N	*P* value (vs CTRL)	Mean ± s.e.m.	N	*P* value (vs CTRL)	
2[1]	D1	CTRL	2.3 ± 0.4	12	--	11.8 ± 1.4	13	--	****
		unc-51	1.7 ± 0.3	12	ns	11.1 ± 1.4	13	ns	****
		atg-18	0.7 ± 0.2	12	ns	3.3 ± 0.9	13	****	ns
		bec-1	2.6 ± 0.4	12	ns	4.2 ± 1.1	13	****	ns
		atg-7	1.8 ± 0.5	12	ns	2.2 ± 1.2	13	****	ns
		atg-13	2.3 ± 0.3	13	ns	10.2 ± 0.9	13	ns	****
		hsf-1	2.1 ± 0.4	12	ns	0.9 ± 0.3	13	****	ns
3	D1	CTRL	3.0 ± 0.6	10	--	18.3 ± 1.5	12		***
		unc-51	3.3 ± 0.8	10	ns	19.8 ± 1.2	12	ns	****
		atg-18	3.4 ± 0.5	10	ns	4.8 ± 1.2	12	****	ns
		bec-1	2.7 ± 0.4	10	ns	5.8 ±1.2	12	****	ns
		hsf-1[2]	5.5 ± 0.8	11	ns	0.7 ± 0.31	12	****	**

Neurons

Exp	Age	RNAi	Control			Heat shock and 2-hour Recovery			*P* value (CTRL vs HS)
			Mean ± s.e.m.	N	*P* value (vs CTRL)	Mean ± s.e.m.	N	*P* value (vs CTRL)	
1[1]	D1	CTRL	24.7 ± 1.1	12	--	31.0 ± 1.6	12		ns
		lgg-1	6.4 ± 0.9	12	****	5.8 ± 0.7	12	****	ns
		unc-51	23.6 ± 1.1	12	ns	15.7 ± 1.7	11	****	*
		atg-18	24.3 ± 1.9	11	ns	21.8 ± 1.5	13	**	ns
		bec-1	29.6 ± 1.8	11	ns	23.0 ± 1.8	11	*	ns
		atg-7	26.7 ± 1.4	11	ns	25.7 ± 1.7	12	ns	ns
		atg-13	27.8 ± 1.3	12	ns	17.3 ± 1.5	12	****	***
		hsf-1	30.8 ± 1.8	12	ns	18.8 ± 1.5	12	****	****
2	D1	CTRL	21.6 ± 0.6	11	--	27.9 ± 1.4	11	--	*
		lgg-1	15.7 ± 1.5	11	ns	17.2 ± 1.2	11	****	ns
		unc-51	13.3 ± 1.1	11	***	15.1 ± 0.8	11	****	ns
		atg-18	13.6 ± 2.0	11	***	12.1 ± 1.2	11	****	ns
		bec-1	17.3 ± 1.0	11	ns	17.6 ± 1.0	11	****	ns
		atg-7	17.8 ± 1.2	11	ns	17.0 ± 1.0	11	****	ns
		atg-13	14.5 ± 0.7	11	**	17.0 ± 1.3	11	****	ns
		hsf-1[2]	20.4 ± 0.7	12	ns	14.6 ± 1.1	11	****	**

(Supplementary Table 5 continued)

Muscle									
Exp	Age	RNAi	Control			Heat shock and 0-hour Recovery			*P* value (CTRL vs HS)
			Mean ± s.e.m.	N	*P* value (vs CTRL)	Mean ± s.e.m.	N	*P* value (vs CTRL)	
1[1]	D1	CTRL	1.5 ± 0.4	13	--	5.3 ± 1.2	12	--	****
		unc-51	1.9 ± 0.4	13	ns	2.9 ± 0.9	11	ns	ns
		atg-18	1.9 ± 0.7	13	ns	1.5 ± 0.5	13	**	ns
		bec-1	2.4 ± 0.8	13	ns	1.7 ± 0.4	13	*	ns
		atg-7	0.7 ± 0.3	13	ns	1.4 ± 0.3	13	**	ns
		atg-13	3.4 ± 0.8	13	ns	1.6 ± 0.5	13	**	ns
		hsf-1	3.1 ± 0.8	13	ns	1.6 ± 0.6	12	**	ns
2	D1	CTRL	2.1 ± 0.5	9	--	5.0 ± 1.0	11	--	*
		unc-51	2.3 ± 0.5	10	ns	2.0 ± 0.4	12	*	ns
		atg-18	1.3 ± 0.6	11	ns	0.9 ± 0.3	11	****	ns
		bec-1	1.7 ± 0.6	10	ns	1.4 ± 0.5	12	***	ns
		hsf-1	2.5 ± 0.6	10	ns	1.7 ± 0.4	11	**	ns
3[2]	D1	CTRL	0.6 ± 0.4	10	--	3.3 ± 0.3	9	--	****
	D1	*hsf-1*	0.9 ± 0.4	8	ns	0.7 ± 0.2	10	****	ns

Supplementary Table 5: GFP::LGG-1-positive punctae were quantified in the hypodermal seam cells, intestine, and muscle of animals expressing *lgg-1p::gfp::lgg-1* (DA2123) or in the neurons of animals expressing *rgef-1p::gfp::lgg-1* (MAH242) raised from hatching (WL: whole-life RNAi) on control bacteria (empty vector, CTRL) or bacteria expressing dsRNA targeting the indicated genes. Animals were maintained under control conditions or subjected to 1 h of hormetic heat shock at 36°C followed by the indicated recovery time. ns: $P > 0.05$, *$P < 0.05$, **$P < 0.01$, ***$P < 0.001$, ****$P < 0.0001$ by two-way ANOVA; Exp: experiment number (in chronological order); s.e.m.: standard error of the mean; N: number of cells or animals analyzed (see Methods). [1]: data depicted in **Supplementary Fig. 3**; [2]: data depicted in **Supplementary Fig. 8.**

Supplementary Table 6: Autophagy measurements in wild-type animals following different durations of heat shock.

Hypodermal seam cells (0-hour Recovery)					
**Exp**	**Age**	**Heat shock**	**Mean ± s.e.m.**	**N**	***P* value**
1	D1	CTRL	0.4 ± 0.1	74	
		15 min	2.7 ± 0.2	64	****
		30 min	3.6 ± 0.3	47	****
		45 min	3.1 ± 0.2	49	****
		60 min	2.7 ± 0.2	49	****
2[1]	D1	CTRL	0.4 ± 0.1	95	
		15 min	1.7 ± 0.1	75	****
		30 min	2.0 ± 0.1	78	****
		45 min	1.9 ± 0.1	82	****
		60 min	1.8 ± 0.1	96	****
**Intestine (4-hour Recovery)**					
**Exp**	**Age**	**Heat shock**	**Mean ± s.e.m.**	**N**	***P* value**
1	D1	CTRL	2.8 ± 1.1	13	
		15 min	3.1 ± 1.1	15	ns
		30 min	4.5 ± 2.4	13	ns
		45 min	14.1 ± 5.7	13	****
		60 min	19.3 ± 4.8	14	****
2[1]	D1	CTRL	3.7 ± 0.3	13	
		15 min	3.9 ± 0.3	11	ns
		30 min	5.0 ± 0.4	12	ns
		45 min	10.4 ± 1.0	12	****
		60 min	14.1 ± 1.2	12	****
**Neurons (2-hour Recovery)**					
**Exp**	**Age**	**Heat shock**	**Mean ± s.e.m.**	**N**	***P* value**
1	D1	CTRL	17.1 ± 0.8	12	
		15 min	21.3 ± 0.6	11	***
		30 min	20.5 ± 0.6	13	**
		45 min	20.1 ± 0.7	10	*
		60 min	20.3 ± 0.8	12	**
2[1]	D1	CTRL	17.8 ± 0.7	12	
		15 min	25.6 ± 0.9	13	****
		30 min	22.9 ± 0.9	12	***
		45 min	24.2 ± 0.7	13	****
		60 min	22.2 ± 0.9	13	**

(Supplementary Table 6 continued)

Muscle (0-hour Recovery)					
**Exp**	**Age**	**Heat shock**	**Mean ± s.e.m.**	**N**	***P* value**
1[1]	D1	CTRL	1.9 ± 0.3	12	
		15 min	5.9 ± 0.9	12	*
		30 min	7.5 ± 0.9	12	***
		45 min	9.2 ± 1.7	10	****
		60 min	6.6 ± 1.1	10	*
2	D1	CTRL	3.3 ± 0.6	12	
		15 min	8.8 ± 1.6	10	**
		30 min	8.1 ± 1.6	10	*
		45 min	7.8 ± 0.9	10	*
		60 min	7.5 ± 1.1	10	*

Supplementary Table 6: GFP::LGG-1-positive punctae were quantified in the hypodermal seam cells, intestine, and muscle of animals expressing *lgg-1p::gfp::lgg-1* (DA2123) and in the neurons of animals expressing *rgef-1p::gfp::lgg-1* (MAH242). Animals were maintained under control conditions or subjected to heat shock at 36°C for the indicated times on day 1 of adulthood followed by the indicated recovery time. ns: $P > 0.05$, *$P < 0.05$, **$P < 0.01$, ***$P < 0.001$, ****$P < 0.0001$ by one-way ANOVA; Exp: experiment number (in chronological order); s.e.m.: standard error of the mean; N: number of cells or animals analyzed (see Methods). [1]: data depicted in **Supplementary Fig. 6**.

Supplementary Table 7: Autophagy measurements in animals overexpressing HSF-1.

Exp	Age	Wild-type		HSF-1 OE		*P* value
**Hypodermal seam cells**						
		Mean ± s.e.m.	N	Mean ± s.e.m.	N	
1	D1	0.7 ± 0.3	171	1.7 ± 0.2	79	***
2[1]	D1	0.6 ± 0.1	163	1.4 ± 0.1	131	****
**Intestine**						
Exp	Age	Wild-type		HSF-1 OE		*P* value
		Mean ± s.e.m.	N	Mean ± s.e.m.	N	
1[1]	D1	2.4 ± 0.3	13	3.8 ± 0.6	13	*
2	D1	5.5 ± 0.6	10	8.3 ± 0.6	10	**
**Neurons**						
Exp	Age	Wild-type		HSF-1 OE		*P* value
		Mean ± s.e.m.	N	Mean ± s.e.m.	N	
1[1]	D1	23.2 ± 1.6	11	29.3 ± 1.6	12	*
2	D1	19.0 ± 2.2	4	25.7 ±1.1	6	*
**Muscle**						
Exp	Age	Wild-type		HSF-1 OE		*P* value
		Mean ± s.e.m.	N	Mean ± s.e.m.	N	
1[1]	D1	0.4 ± 0.7	10	1.2 ± 0.3	10	*
2	D1	0.6 ± 0.2	14	1.5 ± 0.3	15	*

Supplementary Table 7: GFP::LGG-1-positive punctae were quantified in the hypodermal seam cells, intestine, and muscle of animals expressing *lgg-1p::gfp::lgg-1* (MAH236) and *lgg-1p::gfp::lgg-1* + *let-858p::hsf-1* (MAH534) and in the neurons of animals expressing *rgef-1p::gfp::lgg-1* (MAH242) and *rgef-1p::gfp::lgg-1* + *let-858p::hsf-1* (MAH552). **P* < 0.05, ***P* < 0.01, ****P* < 0.001, *****P* < 0.0001 by Student's *t*-test; Exp: experiment number (in chronological order); s.e.m.: standard error of the mean; N: number of cells or animals analyzed (see Methods). [1]: data depicted in **Fig. 2a–d**.

Supplementary Table 8: HSE elements in promoter regions of autophagy-related genes in *C. elegans*.

Gene	Sequence name	Function	Putative HSE 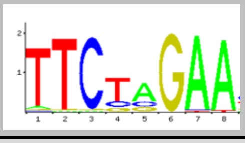	Distance of HSE to start site
asp-1	Y39B6A.20	Lysosomal cathepsin D aspartic protease	TTCTAGAT	-1834 bp
			GTCTAGAA	-1733 bp
			TTCTAGAA	-711 bp
atg-16.1	F02E8.5	Autophagosome elongation	CTCTAGAA	-1196 bp
atg-3	Y55F3AM.4	AuTophaGy (yeast Atg homolog)	TTCTCGAA	-1685 bp
			GTCTAGAA	-852 bp
			TTCTGGAA	-703 bp
			TTCTGGAA	-487 bp
			TTCTGGAA	-45 bp
atg-4.1	Y87G2A.3	Autophagosome elongation	TTCGAGAA	-258 bp
			TTCGAGAA	-177 bp
atg-4.2	ZK792.8	Autophagosome elongation	TTCTGGAA	-1230 bp
			TTCTAGAC	-1033 bp
atg-5	Y71G12B.12	Autophagosome elongation	TTCTCGAA	-534 bp
atg-7	M7.5	Autophagosome elongation	TTCTAGAA	-1406 bp
atg-9	T22H9.2	Phagophore formation	ATCTCGAA	-1956 bp
			TTCTCGAA	-857 bp
			TTCCAGAA	-535 bp
bec-1	T19E7.3	Membrane nucleation	TTCTGGAA	-1906 bp
			TTCTGGAA	-1817 bp
			TTCTAGAT	-1769 bp
			TTCTCGAA	-1468 bp
			TTCCAGAA	-596 bp
C06G3.6	C06G3.6	Hypothetical protein	TTCTAGAA	-65 bp
			TTCCAGAA	-44 bp
C08H9.1	C08H9.1	Putative lysosomal serine carboxypeptidase/CTSA homolog	TTCTCGAA	-1236 bp
			TTCCAGAA	-375 bp
ced-9	T07C4.8	Homolog of the mammalian cell-death inhibitor Bcl-2	TTCCAGAA	-1453 bp
			GTCTAGAA	-44 bp
epg-2	Y39G10AR.10	Ectopic P granule protein	TTCTGGAA	-1888 bp
epg-4	F37C12.2	Ectopic P granule protein	TTCTGGAA	-1828 bp
epg-5	C56C10.12	Ectopic P granule protein	TTCTAGAT	-707 bp
			TTCTCGAA	-473 bp

(**Supplementary Table 8 continued)**

Gene	Sequence name	Function Putative	Putative HSE 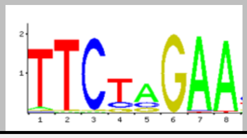	Distance of HSE to start site
epg-6	Y39A1A.1	Ectopic P granule protein	TTCTCGAA	-984 bp
			TTCCAGAA	-945 bp
			TTCTCGAA	-49 bp
			TTCCAGAA	-10 bp
epg-8	Y106G6A.2	Ectopic P granule protein	TTCGAGAA	-1826 bp
			TTCGAGAA	-1552 bp
			TTCGAGAA	-990 bp
epg-9	Y69A2AR.7	Ectopic P granule protein	TTCCAGAA	-1852 bp
			TTCTGGAA	-1112 bp
			CTCTAGAA	-447 bp
			ATCTCGAA	-397 bp
			TTCCAGAA	-238 bp
gpd-2	K10B3.8	Glyceraldehyde-3-phosphate dehydrogenase 2	TTCTCGAA	-1440 bp
			TTCTAGAA	-552 bp
			TTCTCGAA	-364 bp
hlh-30	W02C12.3	TFEB homolog	ATCTAGAA	-1575 bp
			GTCTAGAA	-1200 bp
			GTCTAGAA	-523 bp
let-363	B0261.2	MTOR homolog	TTCTAGAA	-2047 bp
			TTCCAGAA	-2010 bp
			ATCTAGAA	-1553 bp
			TTCCAGAA	-710 bp
			TTCTAGAT	-479 bp
lgg-2	ZK593.6	Autophagosome elongation	TTCTAGAG	-1633 bp
			TTCCAGAA	-1574 bp
			TTCTAGAG	-811 bp
			TTCTAGAG	-272 bp
lgg-3	B0336.8	Autophagosome elongation	ATCTCGAA	-1539 bp
lipl-3	R11G11.14	Lysosomal lipolysis	TTCTAGAA	-1105 bp
lipl-4	K04A8.5	Lysosomal lipolysis	TTCTAGAC	-1006 bp
			TTCTGGAA	-338 bp
			TTCTAGAC	-285 bp
lmp-2	C05D9.2	Lysosomal membrane	TTCTAGAT	-1764 bp
			TTCTAGAA	-813 bp
sdz-30	T04D3.2	SKN-1 Dependent Zygotic transcript	TTCTAGAA	-1534 bp
			TTCTAGAA	-1509 bp

(Supplementary Table 8 continued)

Gene	Sequence name	Function	Putative HSE 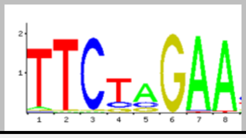	Distance of HSE to start site
sepa-1	M01E5.6	Suppressor of ectopic P granules in autophagy mutant	TTCCAGAA	-18 bp
sqst-1	T12G3.1	Cargo sequestration	TTCTAGAG	-944 bp
			TTCTAGAG	-419 bp
sqst-2	Y40C5A.1	Cargo sequestration	TTCTGGAA	-1391 bp
			TTCTAGAC	-1232 bp
sqst-3	T26H2.5	Cargo sequestration	TTCCAGAA	-1802 bp
sqst-4	Y32B12A.1	Cargo sequestration	TTCTAGAA	-1256 bp
sul-3	C54D2.4	Lysosomal sulfatase	TTCTAGAA	-1253 bp
			TTCTAGAA	-1145 bp
			TTCTCGAA	-256 bp
			TTCTCGAA	-148 bp
T04D3.1	T04D3.1	SEPA-like[2]	TTCTAGAA	-409 bp
			TTCTAGAA	-384 bp
vet-2	C35E7.1	SEPA-like[2]	GTCTAGAA	-793 bp
vet-6	F44F1.7	SEPA-like[2]	TTCTAGAT	-327 bp
			GTCTAGAA	-48 bp
			TTCCAGAA	-21 bp
vha-15	T14F9.1	Vacuolar pH	TTCTGGAA	-1009 bp
			TTCTAGAA	-717 bp
			TTCGAGAT	-574 bp
			TTCTGGAA	-157 bp
vps-11	R06F6.2	Lysosomal Fusion	TTCCAGAA	-1813 bp
vps-15	ZK930.12	Lysosomal Fusion	TTCCAGAA	-1690 bp
			TTCGAGAA	-452 bp
vps-18	W06B4.3	Lysosomal Fusion	TTCTCGAA	-1867 bp
			TTCTGGAA	-1301 bp
			TTCTCGAA	-1180 bp
			TTCTGGAA	-1040 bp
vps-34	B0025.1	Membrane nucleation	TTCGAGAA	-1995 bp
			TTCTCGAA	-540 bp
			TTCTAGAA	-272 bp
ZK1053.4	ZK1053.4	SEPA-1-like[2]	ATCTAGAA	-1473 bp
			GTCTAGAA	-1391 bp

Supplementary Table 8: Putative heat shock elements (HSE) in promoter regions of *C. elegans* autophagy-related genes (i.e., in the 2000 base pairs [bp] upstream of the start codon). The *C. elegans* HSE motif profile (Ref [1]) was identified using JASPAR. [2]: selective turnover of P granules.

Supplementary Table 9: Thermorecovery analyses of *hlh-30* mutants after hormetic heat shock.

Exp	Strain	Heat shock conditions	Control			Heat shock and 0-hour Recovery		*P* value (CTRL vs HS)
			% alive ± s.e.m.	N	*P* value (vs WT)	% alive ± s.e.m.	N	
2	WT	HS: 30 min, 36°C, d1 TR: d3, 8 h	88 ± 3	96		95 ± 2	110	ns
	hlh-30(tm1978)		8 ± 2	115	****	20 ± 3	99	*
3[1]	WT	HS: 30 min, 36°C, d1 TR: d3, 8 h	12 ± 4	101		39 ± 4	112	**
	hlh-30(tm1978)		6 ± 3	106	ns	10 ± 2	93	ns
4	WT	HS: 30 min, 36°C, d1 TR: d3, 8 h	44 ± 4	140		67 ± 3	114	***
	hlh-30(tm1978)		8 ± 2	115	****	11 ± 2	114	ns
5	*WT*	HS: 30 min, 36°C, d1 TR: d4, 8 h	48 ± 5	131		64 ± 2	141	*
	hlh-30(tm1978)		24 ± 5	155	**	39 ± 3	153	*

Supplementary Table 9: Survival analysis of wild-type (WT, N2) and *hlh-30(tm1978)* mutants (JIN1375). Animals were maintained under control conditions or subjected to 30 min of hormetic heat shock (HS: 36°C) on day 1 (d1) of adulthood. Thermorecovery (TR) was tested on the indicated days of adulthood by incubating animals at 36°C for 6–9 h followed by ~20 h of recovery. Exp: experiment number (in chronological order). The mean ± s.e.m. percentage survival of experimental and control groups were analyzed by two-way ANOVA. ns: *P* > 0.05, **P* < 0.05, ***P* < 0.01, ****P* < 0.001; N: number of animals analyzed (see Methods). [1]: data depicted in **Supplementary Fig. 9d.**

Supplementary Table 10: Lifespan analyses of *hlh-30* mutants after hormetic heat shock.

Exp	Strain	Control				Heat shock		CTRL vs HS	
		MLS (days)	N	% MLS	*P* value (vs WT)	MLS (days)	N	% MLS	*P* value
1	WT	14.4	107/120			21.4	85/121	+48.6%	<0.0001
	hlh-30(tm1978)	15.3	90/122	+6.3%	0.5	15.5	85/124	+1.3%	0.5
2[1]	WT	17.6	124/126			21.7	114/120	+23.3%	<0.0001
	hlh-30(tm1978)	12.5	118/121	-29.0%	<0.0001	11.6	115/120	-7.2%	0.33
3	WT	15.4	125/129			20.8	114/121	+35.1%	<0.0001
	hlh-30(tm1978)	9.8	116/124	-36.4%	<0.0001	11.7	117/132	+19.4%	0.0001

Supplementary Table 10: Lifespan analysis of wild-type (WT, N2) and *hlh-30(tm1978)* (JIN1375) mutants. Animals were maintained under control conditions or subjected to 30 min of hormetic heat shock (HS: 36°C) on day 1 (d1) of adulthood. Exp: experiment number (in chronological order). MLS: mean lifespan; N: observed deaths/total number of animals; % MLS: percentage change in lifespan compared with control; *P*-values calculated by log-rank test. [1]: data depicted in **Supplementary Fig. 9e**.

Supplementary Table 11: Thermorecovery analyses of animals overexpressing HSF-1.

Exp	Strain	RNAi	% alive	N	*P* value
1	HSF-1 OE	CTRL (WL)	35 ± 6	127	
	HSF-1 OE	*unc-51* (WL)	16 ± 4	101	*
	HSF-1 OE	*atg-18* (WL)	5 ± 2	107	***
	HSF-1 OE	*bec-1* (WL)	3 ± 2	101	***
	HSF-1 OE	*lgg-1* (WL)	8 ± 3	107	***
	WT	CTRL (WL)	6 ± 2	122	***
2	HSF-1 OE	CTRL (WL)	54 ± 1	113	
	HSF-1 OE	*unc-51* (WL)	29 ± 7	209	**
	HSF-1 OE	*atg-18* (WL)	24 ± 1	137	**
	HSF-1 OE	*bec-1* (WL)	34 ± 5	220	*
	HSF-1 OE	*lgg-1* (WL)	19 ± 5	120	***
	WT	CTRL (WL)	30 ± 3	130	**
3[1]	HSF-1 OE	CTRL (AO)	42 ± 2	142	
	HSF-1 OE	*unc-51* (AO)	26 ± 5	100	*
	HSF-1 OE	*atg-18* (AO)	20 ± 2	145	**
	HSF-1 OE	*bec-1* (AO)	18 ± 5	123	**
	HSF-1 OE	*lgg-1* (AO)	23 ± 3	154	*
	WT	CTRL (AO)	29 ± 7	91	ns
4	HSF-1 OE	CTRL (AO)	46 ± 3	116	
	HSF-1 OE	*unc-51* (AO)	8 ± 1	122	****
	HSF-1 OE	*atg-18* (AO)	5 ± 1	91	****
	HSF-1 OE	*bec-1* (AO)	17 ± 5	123	***
	HSF-1 OE	*lgg-1* (AO)	14 ± 5	131	***
	WT	CTRL (AO)	31 ± 4	108	ns

Supplementary Table 11: Survival analysis of wild-type (N2) and HSF-1–overexpressing (HSF-1 OE) animals (*hsf-1p::hsf-1::gfp,* MAH365) fed control bacteria (empty vector, CTRL) or bacteria expressing dsRNA targeting the indicated autophagy genes from hatching (whole-life RNAi; WL) or from day 1 of adulthood (adult-only RNAi; AO). Animals were incubated at 36°C for 7–8 h on day 3–4 of adulthood followed by recovery for ~20 h. Exp: experiment number (in chronological order). The mean ± s.e.m. percentage survival of experimental and control groups were analyzed using Student's *t*-test or one-way ANOVA. ns: $P > 0.05$, *$P < 0.05$, **$P < 0.01$, ***$P < 0.001$; N: number of animals analyzed (see Methods). [1]: data depicted in **Fig. 3b.**

Supplementary Table 12: Lifespan analyses of animals overexpressing HSF-1.

Exp	RNAi	Wild-type				HSF-1 Overexpressor				% MLS	*P* value
		MLS (days)	N	% MLS	*P* value	MLS (days)	N	% MLS	*P* value		
1	CTRL	16.9	113/121			19.2	101/121			+13.8%	0.006
	atg-18	14.2	110/122	-15.6%	<0.0001	12.2	111/124	-36.7%	<0.0001		
2	CTRL	16.3	104/121			22.9	99/123			+40.5%	<0.0001
	atg-18	13.5	100/121	-17.2%	<0.0001	14.7	117//125	-35.8%	<0.0001		
	atg-13	18.0	112/112	+10.4%	0.03	15.0	111/126	-34.5%	<0.0001		
	lgg-1	18.4	91/120	+12.9%	0.04	17.4	118/128	-24.0%	<0.0001		
3	CTRL	16.0	93/124			19.4	107/148			+21.3%	<0.0001
	lgg-1	15.6	97/128	-2.5%	0.6	15.2	164/177	-24.6%	<0.0001		
	atg-13	17.1	113/130	+6.9%	0.2	16.2	147/168	-17.0%	0.003		
4[1]	CTRL	18.1	113/130			23.0	121/143			+27.2%	<0.0001
	unc-51	18.3	128/138	+1.5%	0.9	15.4	133/139	-33.1%	<0.0001		
	bec-1	16.7	123/133	-7.4%	0.02	16.3	140/153	-29.1%	<0.0001		
	lgg-1	16.7	109/142	-7.6%	0.02	14.9	147/153	-35.2%	<0.0001		

Supplementary Table 12: Lifespan analysis of wild-type animals and HSF-1–overexpressing animals *hsf-1p::hsf-1::gfp + rol-6.* Experiment 1 used strain EQ87; all other experiments used MAH365 (MAH365 is EQ87 4x outcrossed to Hansen lab N2). Animals were fed from day 1 of adulthood with control bacteria (empty vector, CTRL) or bacteria expressing dsRNA targeting the indicated autophagy genes. Exp: experiment number (in chronological order). MLS: mean lifespan. N: observed deaths/total number of animals subjected to RNAi. % MLS: percentage change in lifespan compared with control. ns: $P > 0.05$, *$P < 0.05$, **$P < 0.01$, ***$P < 0.001$, ****$P < 0.0001$ by log-rank test. [1]: data depicted in **Fig. 3f–h.**

Supplementary Table 13: Lifespan analyses of PolyQ-expressing animals after hormetic heat shock.

Exp	Strain	Control				Heat shock		CTRL vs HS	
		MLS (days)	N	% MLS	*P* value (vs WT)	MLS (days)	N	% MLS	*P* value
1	*vha-6p::Q44::yfp*	16.8	107/127			18.5	108/134	+10.1%	0.004
2	WT	17.2	122/127			21.2	105/126	+23.3%	<0.0001
	vha-6p::Q44::yfp[1]	15.2	134/151	-11.6%	0.07	19.3	115/124	+27.0%	<0.0001
	rgef-1p::Q40::yfp	11.9	115/125	-30.8%	<0.0001	15.4	98/124	+29.4%	<0.0001
3	WT	15.4	111/123			17.5	102/120	+13.6%	0.0006
	vha-6p::Q44::yfp	14.0	133/135	-9.1%	0.005	18.1	104/139	+29.3%	<0.001
	rgef-1p::Q40::yfp[2]	9.4	118/130	-39.0%	<0.0001	12.4	94/124	+31.9%	0.0001
4	WT	16.4	129/130			20.2	119/123	+23.2%	<0.0001
	vha-6p::Q44::yfp	14.7	129/130	-10.4%	0.003	18.2	119/128	+23.8%	<0.0001
	rgef-1p::Q40::yfp[4]	9.2	112/127	-43.9%	<0.0001	15.5	79/127	+68.5%	<0.0001

Supplementary Table 13: Lifespan analysis of wild-type and the indicated mutant and transgenic animals maintained under control conditions or subjected to hormetic heat shock (HS: 36°C) for 30 min on day 1 of adulthood. MLS: mean lifespan; N: observed deaths/total number of animals; % MLS: percentage change in lifespan compared with control. *P*-values calculated by log-rank test. [1]: data depicted in **Fig. 4e**; [2]: data depicted in **Supplementary Fig. 13c**.

Supplementary Table 14: *C. elegans* strains used in this study.

Published strains used in this study		
**Name**	**Genotype**	**Strain origin**
N2	Wild-type (WT)	Hansen lab, originated from Kenyon lab
AM101	*rmIs110[rgef-1p::polyQ40::YFP]*	Morimoto lab)
AM446	*rmIs223[C12C8.1p::GFP; rol-6(su1006)]*	Morimoto lab
BC13209	*dpy-5(e907) I; sEx13209 [atg-18p::GFP + dpy-5(+)]*	Baillie lab
CB1301	*unc-54(e1301) I (ts)*	CGC
CB1402	*unc-15(e1402) I (ts)*	CGC
CF1553	*muIs84[sod-3p::gfp]*	Kenyon lab
CL2166	*dvIs19[gst-4p::gfp::NLS] III*	CGC
CX51	*dyn-1(ky51) X (ts)*	CGC
DA2123	*adIs2122[lgg-1p::gfp::lgg-1 + rol-6(su1006)]*	Avery lab
DG718	*Ex[let-858p::hsf-1 + rol-6(su1006)]*	Gidalevitz lab
EQ87	*iqIs28[hsf-1p::hsf-1::gfp + rol-6(su1006)]*	Hsu lab
GF80	*dgEx80[vha-6p::Q44::yfp + rol-6(su1006)]*	CGC
HZ1330	*bpEx50(atg-16.2p::gfp + unc-76)*	Zhang lab
JIN1375	*hlh-30(tm1978) IV*	Irazoqui lab
LD1171	*ldIs3[gcs-1p::gfp + rol-6(su1006)]*	CGC
MAH235	*sqIs19[hlh-30p::hlh-30::gfp + rol-6(su1006)]*	Hansen lab
MAH236	*sqIs13[lgg-1p::gfp::lgg-1 + odr-1p::rfp]*	Hansen lab
MAH242	*sqIs24[rgef-1p::gfp::lgg-1 + unc-122p::rfp]*	Hansen lab
RD202	*unc-119(ed3) III; ppIs??[unc-119(+); lgg-1p::gfp::lgg-1(G116A)]*	Legouis lab
SD551	*let-60(ga89) IV (ts)*	CGC
SJ4005	*zcIs4[hsp-4p::gfp] V*	CGC
SJ4100	*Iszc13[hsp-6p::gfp]*	CGC
TJ375	*gpIs1[hsp-16.2p::gfp]*	CGC
**Strains generated in this study**		
**Name**	**Genotype**	**Comments**
MAH215	*sqIs11[lgg-1p::mcherry::gfp::lgg-1 + rol-6(su1006)]*	Microinjection of pMH878 *(lgg-1p::mcherry::gfp::lgg-1)* plus pRF4 (*rol-6*) into N2 (to generate MAH167). Gamma-irradiation of MAH167, and 4x outcrossed to N2. Chang JT et al., manuscript in revision
MAH325	*dpy-5(e907) I/?; sIs10729[sqst-1p::GFP + dpy-5(+)]*	BC12921 4x outcrossed to N2
MAH365	*iqIs28[hsf-1p::hsf-1::gfp + rol-6(su1006)]*	EQ87 4x outcrossed to N2
MAH471	*unc-15(e1402) I (ts)*	CB1402 4x outcrossed to N2
MAH477	*dyn-1(ky51) X (ts)*	CX51 4x outcrossed to N2
MAH478	*let-60(ga89) IV (ts)*	SD551 4x outcrossed to N2
MAH534	*sqIs13[lgg-1p::gfp::lgg-1 + odr-1p::rfp]; Ex[let-858p::hsf-1 + rol-6(su1006)]*	MAH236 x DG718
MAH552	*sqIs24[rgef-1p::gfp::lgg-1 + unc-122p::rfp]; Ex[let-858p::hsf-1 + rol-6(su1006)]*	MAH242 x DG718
MAH575	*iqIs28[hsf-1p::hsf-1::gfp + rol-6(su1006)]; dgEx80[vha-6p::Q44::YFP + rol-6(su1006)]*	GF80 x MAH365
MAH602	*sqIs61[vha-6p::Q44::YFP + rol-6(su1006)]*	Gamma-irradiation of GF80 and 6x outcrossed to N2

Supplementary Table 15: Sequences of quantitative RT-PCR primers used in this study.

Gene	Primer sequence 5′ → 3′	
**Experimental genes**		
unc-51	Fwd	ACA AAT CCC TGT CGT TCC AG
	Rev	AAT GAG CCG TTG GAT AAT GC
atg-9	Fwd	GGC CGC CAT CCA CTC ATC GG
	Rev	GGC CGC CAT CCA CTC ATC GG
atg-18	Fwd	AAA TGG ACA TCG GCT CTT TG
	Rev	TGA TAG CAT CGA ACC ATC CA
bec-1	Fwd	TCGAGCTCCCACTCTTTGGCG
	Rev	TGA CAC CAT TGT CAA CCA GTG
lgg-1	Fwd	ACC CAG ACC GTA TTC CAG TG
	Rev	ACG AAG TTG GAT GCG TTT TC
sqst-1	Fwd	TGG CTG CTG CAT CAT CCG CT
	Rev	TCA ATC GTG CCG AGA CCG GG
vps-11	Fwd	TCC GCT TGT CGT CCT GGA GC
	Rev	TCA CAC GCC GAG CAC TTG GT
lmp-1	Fwd	ATCCGCCACCGCTTCGCATT
	Rev	TCGAGCTCCCACTCTTTGGCG
vha-15	Fwd	CGA GGT TCG TTC CGG ACG TCT T
	Rev	CCT CGG CAG TCA GGA GAC GC
vha-16	Fwd	AGG CGC TGA CTC GCG GAC TT
	Rev	TGG TCT CTG GTG AAG AGT TCC GGT G
hsp-70 (C12C8.1)	Fwd	ACT CAT GTG TCG GTA TTT ATC
	Rev	ACG GGC TTT CCT TGT TTT
hsp-16.1	Fwd	GTC ACT TTA CCA CTA TTT CCG TCC AGC TCA ACG TTC
	Rev	CAA CGG GCG CTT GCT GAA TTG GAA TAG ATC TTC C
hsp-16.2	Fwd	ACT TTA CCA CTA TTT CCG TCC AGC
	Rev	CCT TGA ACC GCT TCT TTC TTT G
aip-1	Fwd	GGC GGA GTT CCC AAA TCT CGG AAA GCA CTG TG
	Rev	GGT GCA GTT GGA ATT GGA ATT TCT TGT TTG ATG C
gst-4	Fwd	CTC AAT GTG CCT TAC GAG G
	Rev	CGA ATT GTT CTC CAT CGA CTT G
gcs-1	Fwd	GTC GAT TGA TGA GAT TAT CAA TGG
	Rev	CAA AGT ATT AAT CTC TCC AGT TGC
sod-3	Fwd	GCA TCA TGC CAC CTA CGT G
	Rev	CTG GTT GGA GAG CAA TTG CTT C
hsp-6	Fwd	GAC GCT AAT GGT ATC GTG
	Rev	GAT AAC CTC GAC GAG CTC
hsp-3	Fwd	GAC CAA GTA TGG AAC CAT TAT TG
	Rev	GAG AAA GCA ACG TAG GAT G
hlh-30	Fwd	CTC ATC GGC CGG CGC TCA TC
	Rev	AGA ACG CGA TGC GTG GTG GG

(Supplementary Table 15 continued)

Gene	Primer sequence 5′ → 3′	
**Experimental genes**		
ama-1	Fwd	TGG AAC TCT GGA GTC ACA CC
	Rev	CAT CCT CCT TCA TTG AAC GG
pmp-3	Fwd	GTTCCCGTGTTCATCACTCAT
	Rev	ACACCGTCGAGAAGCTGTAGA
cdc-42	Fwd	CTGCTGGACAGGAAGATTACG
	Rev	CTCGGACATTCTCGAATGAAG
nhr-23	Fwd	CAG AAA CAC TGA AGA ACG CG
	Rev	CGA TCT GCA GTG AAT AGC TC

3. Supplementary References

1. GuhaThakurta, D. et al. Identification of a novel cis-regulatory element involved in the heat shock response in Caenorhabditis elegans using microarray gene expression and computational methods. *Genome Research* 12, 701–12 (2002).
